# A small molecule binding HMGB1 inhibits caspase-11-mediated lethality in sepsis

**DOI:** 10.1038/s41419-021-03652-5

**Published:** 2021-04-14

**Authors:** Xiangyu Wang, Zhaozheng Li, Yang Bai, Rui Zhang, Ran Meng, Fangping Chen, Haichao Wang, Timothy R. Billiar, Xianzhong Xiao, Ben Lu, Yiting Tang

**Affiliations:** 1 Department of Hematology and Critical Care Medicine, The 3rd Xiangya Hospital, Central South University, Changsha, 410000 PR China; 2 The Feinstein Institute for Medical Research, Northwell Health, 350 Community Drive, Manhasset, NY 11030 USA; 3 Department of Surgery, University of Pittsburgh Medical Center, Pittsburgh, PA 15213 USA; 4 Key Laboratory of sepsis translational medicine of Hunan, Central South University, Changsha, Hunan 410000 PR China; 5 Department of Physiology, School of Basic Medical Science, Central South University, Changsha, Hunan 410000 PR China

**Keywords:** Cell death and immune response, Sepsis

## Abstract

Caspase-11, a cytosolic lipopolysaccharide (LPS) receptor, mediates lethal immune responses and coagulopathy in sepsis, a leading cause of death worldwide with limited therapeutic options. We previously showed that over-activation of caspase-11 is driven by hepatocyte-released high mobility group box 1 (HMGB1), which delivers extracellular LPS into the cytosol of host cells during sepsis. Using a phenotypic screening strategy with recombinant HMGB1 and peritoneal macrophages, we discovered that FeTPPS, a small molecule selectively inhibits HMGB1-mediated caspase-11 activation. The physical interaction between FeTPPS and HMGB1 disrupts the HMGB1-LPS binding and decreases the capacity of HMGB1 to induce lysosomal rupture, leading to the diminished cytosolic delivery of LPS. Treatment of FeTPPS significantly attenuates HMGB1- and caspase-11-mediated immune responses, organ damage, and lethality in endotoxemia and bacterial sepsis. These findings shed light on the development of HMGB1-targeting therapeutics for lethal immune disorders and might open a new avenue to treat sepsis.

## Introduction

Sepsis, an infection-induced critical illness with multiple organ dysfunctions, is a leading cause of hospital mortality^1^. Elevated levels of circulating lipopolysaccharide (LPS), the major cell-wall component of gram-negative bacteria, are often encountered in sepsis and removal of LPS is beneficial to patients with sepsis^1,2^. Early works establish that LPS stimulates robust production of proinflammatory cytokines, such as tumor necrosis factor (TNF), by signaling through the toll-like receptor 4 (TLR4)/myeloid differentiation factor-2 (MD2) complex^3^. However, targeting the TLR4-MD2 pathway fails to improve the outcome of sepsis in clinical trial^3^. Neutralizing TNF by monoclonal antibody even promotes the mortality in septic patients^4^. These observations raise a possibility that LPS or gram-negative bacteria might cause death through non-TLR4 pathways.

Caspase-11 is an intracellular LPS receptor^5^. Upon activation, it enzymatically cleaves gasdermin D (GSDMD) into membrane pore-forming peptides, leading to a lytic form of cell death, termed pyroptosis, or a hyperactive state of macrophages that release interleukin-1β (IL-1β) without undergoing cell death^6,7^. Pyroptosis leads to robust production of eicosanoids, such as leukotriene B4, which leads to vascular fluid loss and induces shock^8^. GSDMD activation also mediates the externalization of phosphatidylserine and the release of procoagulant microparticles, both of which contribute to the development of disseminated intravascular coagulation (DIC)^9^. The occurrence of DIC promotes multiple organ dysfunctions and markedly increases the mortality in sepsis^10^. Importantly, genetic deletion of Caspase-11 or GSDMD prevents DIC and confers substantial protection during lethal endotoxemia and bacterial sepsis^9^. As caspase-11 locates in the cytoplasm, extracellular LPS is unable to activate caspase-11 unless being delivered into the cytosol of host cells^11^. We recently show hepatocyte-released high mobility group box 1 (HMGB1) could deliver circulating LPS into the cytosol and is required for caspase-11-dependent lethality in endotoxemia and bacterial sepsis^11,12^. In this scenario, HMGB1 physically interacts with LPS and targets its uptake into the lysosomes via the receptor for advanced glycation end-products (RAGE)^11^. HMGB1 in lysosomes permeabilizes the phospholipid bilayer in the acidic environment leading to the release of LPS into the cytosol and caspase-11 activation^11^. Deletion of HMGB1 in hepatocytes or neutralizing HMGB1 with monoclonal antibodies phenocopies caspase-11- or GSDMD-deficiency in endotoxemia and bacterial sepsis^11,12^.

In the light of these findings, we designed a phenotypic screening strategy with recombinant HMGB1, which artificially delivers extracellular LPS into the cytosol of cultured mouse peritoneal macrophages^11^. This cell-based system allows us to screen thousands of different small molecules in a short time and to find the candidates that might effectively inhibit the activation of HMGB1-caspase-11-GSDMD pathway in sepsis. In current study, we show that FeTPPS inhibits HMGB1-mediated caspase-11 activation without affecting global inflammatory responses. Administration of FeTPPS significantly attenuates HMGB1- and caspase-11–mediated GSDMD cleavage, organ damage, and lethality in endotoxemia and bacterial sepsis. Mechanistically, FeTPPS disrupts the HMGB1-LPS binding and decreases the capacity of HMGB1 to induce lysosomal rupture, leading to the diminished cytosolic delivery of LPS and the activation of caspase-11. Together, these findings support the notion that HMGB1 is a drug-target in lethal immune disorders and might bring potential therapeutics for the treatment of sepsis.

## Materials and methods

### Mice

*Casp11*^*−/−*^ mice were purchased from Jackson Laboratory. *NLRP3*^*−/−*^ mice were provided by Prof. Rongbin Zhou. In current study, we use WT littermates as the controls for the transgenic mice. *Hmgb1*^*fl/fl*^ mice were prepared as previously^11^.

Animals were housed in a specific pathogen-free environment at the Department of Laboratory Animals of Central South University. All experimental animal protocols were approved by the Institutional Animal Care and Use Committees of Central South University.

### Antibodies and reagents

Antibodies used in this study include mouse IL-1α (Abcam, ab7632), IL-1β (R&D system, AF-401-NA), GSDMD (Abcam, ab209845), caspase-11 (Novus Biologicals, NB120), caspase-1 (Abcam, ab179515), HMGB1 (Abcam, ab79823), LAMP1 (eBioscience, 14-1071-85), Na+/K+ ATPase (Novus Biologicals, NB300-146), Rab7 (Cell Signaling Technologies, #9367S), cathepsin D (Abcam ab75852), *E. coli* LPS antibodies (Abcam, ab35654), and β-actin (Cell Signaling Technology Inc, #3700s). Other antibodies include human GSDMD (Abcam, ab210070), cleaved N-terminal GSDMD (ab215203), caspase-4 (MBL International, M029-3), IL-1α (Abcam, ab206410), and IL-1β (Abcam, ab9722).

Recombinant HMGB1 protein were kindly provided by Dr. Kevin J. Tracey and T.R.B. Ultrapure LPS (*E. coli* O111:B4, tlrl-3pelps), Nigericin (tlrl-nig), ATP (tlrl-atp), MSU Crystals (tlrl-msu), and Nano-SiO2 (tlrl-sio) were purchased from InvivoGen. DQ ovalbumin (D12053) was purchased from Invitrogen. FeTPPS was purchased from BioVision (2065). Protoporphyrin IX (PIX) was purchased from Selleck (S5145), N-acetyl-l-cysteine (NAC A9165), 1400W dihydrochloride (W4262), N-nitro-l-argininemethylester (L-NAME) (483125), S-methylisothio-urea sulfate (SMT) (M84445), mercaptoethylguanidine (MEG) (M9940), Acridin-e orange hemi(zinc chloride) salt (A6014), Digitonin (D141) were obtained from sigma. Recombinant mouse LPS-binding protein (LBP) (6635-LP-025/CF) was from R&D Systems Inc.

### Endotoxemia model

Male mice aged 8–10 weeks (25–30 g) were randomly chosen and injected intraperitoneally with 25 mg/kg LPS (*E. coli* O111:B4, Sigma), FeTPPS was administered intraperitoneally (6 mg/kg) 1 h prior to LPS challenge. Mice were monitored 5 times daily for a total of 5 days or sacrificed at 16–18 h after LPS injection.

### CLP bacterial sepsis model

Polymicrobial sepsis was induced by cecal ligation and puncture (CLP). Experiments were carried out under pathogen-free conditions with randomly chosen littermates of the same sex, matched by age and body weight. The skin was disinfected with a 2% iodine tincture. Laparotomy was performed under 2% isoflurance (Piramal Critical Care) with oxygen. The cecum was 75% ligated and punctured twice with an 18-gauge needle. Mice were then received a subcutaneous injection of warm sterile saline (1 mL) immediately after surgery for fluid resuscitation. FeTPPS was administrated 1 h before (6 mg/kg) and 2 h after (4 mg/kg) CLP. 16–18 h after CLP, mice were sacrificed to collect serum samples. For survival experiments, mice were monitored for 7 consecutive days after CLP.

### Macrophage cultures and stimulation

Mice in age 8–12 weeks were injected with 3 mL of sterile 3% thioglycollate broth.

After 72 h of thioglycollate broth injection, mice were sacrificed and peritoneal macrophages were harvested. Mouse macrophages were stimulated with LPS + HMGB1 in the presence or the absence of FeTPPS or other compounds. Cell lysates and supernatants were collected 16 h later for western blot, ELISA, or LDH assay.

### Necrotic cell lysate preparation

Wild-type (*Hmgb1*^+/+^) and *Hmgb1*^−/−^ mouse embryonic fibroblasts (MEFs) were purchased from HMGBiotech, Inc. Necrotic Cells lysate were prepared as described previously^11^. The ratio of necrotic cells to macrophages in Fig. 2D is 1:1.

### LPS transfection

Macrophages were seeded into the six-well transfection plate (Bimake) in a total volume of 2 mL (2 × 10^6^ cells/well). Cells were priming with 1 μg/mL of Pam3CSK4 for 4 h in RPMI medium 1640 (Gibco) and transfected with ultrapure LPS or ultrapure LPS + FeTPPS or FeTPPS alone.

### Multiplex cytokine assay

Concentrations of different cytokines were determined in the supernatant of mouse peritoneal macrophages by using the bead-based LEGENDplex assay (Biolegend, 740150) according to the manufacturer’s protocol.

### Isolation of cytosol fraction and LPS activity assay

Subcellular fractionation of mouse peritoneal macrophages was conducted by a digitonin-based fractionation method as described previously with modifications^13^. A total of 4 × 10^6^ cells were stimulated with LPS (1 μg/mL) alone or LPS (1 μg/mL) + HMGB1 (400 ng/mL) in the presence or the absence of FeTPPS (1 μM). After 2 h of stimulation, cells were washed with sterile cold PBS three times and subsequently treated with 300 µl of 0.005% digitonin extraction buffer on ice for 10 min to collect the cytosol. The residual cell fraction was collected in 300 µl of 0.1% CHAPS buffer as described previously (Vanaja et al. 2016)^13^. Western blots for Na^+^/K^+^ ATPase, Rab7, LAMP1, and β-actin were performed to confirm the purity of cytosol fraction.

### Proximity-ligation assay

Interaction between LPS and caspase-11 or HMGB1 was analyzed using a proximity-ligation assay (PLA) kit (Sigma 92008). Cells were cultured in a six-well glass dish with RPMI medium 1640, primed for 4 h with LPS (100 ng/mL) and stimulated with LPS (5 μg/mL) alone or LPS (5 μg/mL) + HMGB1 (10 μg/mL) in the presence or the absence of FeTPPS (1 μM) for 2 h. After wash with PBS three times, cells were fixation with 4% formaldehyde and permeabilization with 0.1% Triton, and incubated over night with primary antibodies against LPS (mouse monoclonal 2D7/1), caspase-11 (rat monoclonal 17D9) or HMGB1 (rabbit monoclonal EPR3507). Then the PLA was conducted according to the manufacturer’s instructions. Images were taken using a Nikon Ni-U microscope and quantified using Image-J software.

### Mouse HMGB1 homologous modeling and docking calculation

Mouse HMGB1 homology modeling was analyzed with the ModWeb Server and the MODELLER program. Box-A (PDB: 4QR9) domain and box-B (PDB: 1HMF) domain from crystal structure of mouse HMGB1 were used as template structure to match the full length of HMGB1 (Figure S10). Small molecule (FeTPPS/PIX) docking was done by AutoDock with AutoDockTools. Molecular graphics was prepared by PyMOL.

### Surface plasmon resonance

The equilibrium-binding constant (KD) of FeTPPS or PIX and HMGB1 was determined by Open SPR conducted using a BIAcore4000 instrument (BIAcore). All the steps were performed according to the previously described protocol^14^.

### Whole-cell patch-clamp recording

Whole-cell current recordings were performed in an EPC10 USB patch-clamp platform (HEKA Elektronik, Lambrecht, Germany). The recording pipets were prepared from glass capillaries (thickness = 0.225 mm) with a PC-10 puller (NARISHIGE, Tokyo, Japan), and the pipet resistance was controlled to be 1.5–3 MΩ. For recording Kv currents, the bath solution contained (in mM): 140 mM KCl, 2.5 mM MgCl2, 10 mM HEPES, and 11 mM EGTA (pH7.2), the external bath solution contained the following: 150 mM NaCl, 5 mM KCl, 25 mM CaCl2, 1 mM MgCl2, 10 mM HEPES, and 10 mM D-glucose, adjusted to pH 5.0. Unless otherwise indicated, all chemicals were products of Sigma-Aldrich (Sigma-Aldrich, St. Louis, MO, USA). After breaking in, the serial resistance was controlled to be less than 10 MΩ, the voltage error was minimized by using 80% serial resistance compensation, and the speed value for compensation was 10 µs. To minimize the fast capacitance, only the tip of the pipet was filled with pipet solution, and the artificial capacitance effect was canceled by using the computer-controlled circuit of the amplifier. Data were acquired by the PatchMaster software and analyzed by Sigmaplot 10.0, Igor Pro 6.10A, and Graphpad Prism 7.01.

### Flow cytometry

For lysosomal rupture measuring, macrophage cells were incubated with 1 µg/mL acridine orange for 15 min and then stimulated as indicated. After treatment, cells were collected and quickly transferred on ice for FACS analysis. Lysosomal rupture can be assessed by loss of emission at 600–650 nm using flow cytometry as described previously^15^.

### RNAi knockdown

For Casp4 siRNA Knockdown, PMA-primed THP-1 cells were seeded in 12-well plates at 5 × 10^5^ cells/well. The siRNA target sequences are TCTACACTATAGTCCAGACCC (CASP4-1), GTCTGGACTATAGTGTAGATG (CASP4-2), and CGTACGCGGAATACTTCGA (control), which were described previously^5^. The knockdown efficiency was examined by western blot using the corresponding antibodies.

### Statistical analysis

All data were analyzed using GraphPad Prism software (version 7.01), (GraphPad Software, Inc., La Jolla, CA, USA). All values are presented as the mean ± SD and representative of three independent experiments. All data met the assumptions of the tests (e.g., normal distribution). Student’s *t*-test was used for comparison between two groups. One-way ANOVA followed by post hoc Bonferroni test were used for multiple comparisons. Survival data were analyzed using the log-rank test. A *p* value < 0.05 was considered statistically significant for all experiments. One asterisk, two asterisks, three asterisks, and four asterisks indicate *p* < 0.05, *p* < 0.01, *p* < 0.001, and *p* < 0.0001, respectively. No samples or animals were excluded. No statistical methods were used to predetermine sample sizes. Sample sizes were similar to those generally employed in the field.

## Results

### Phenotypic screening strategy identifies FeTPPS as a potential selective inhibitor of the HMGB1-caspase-11 pathway

**Fig. 1:**
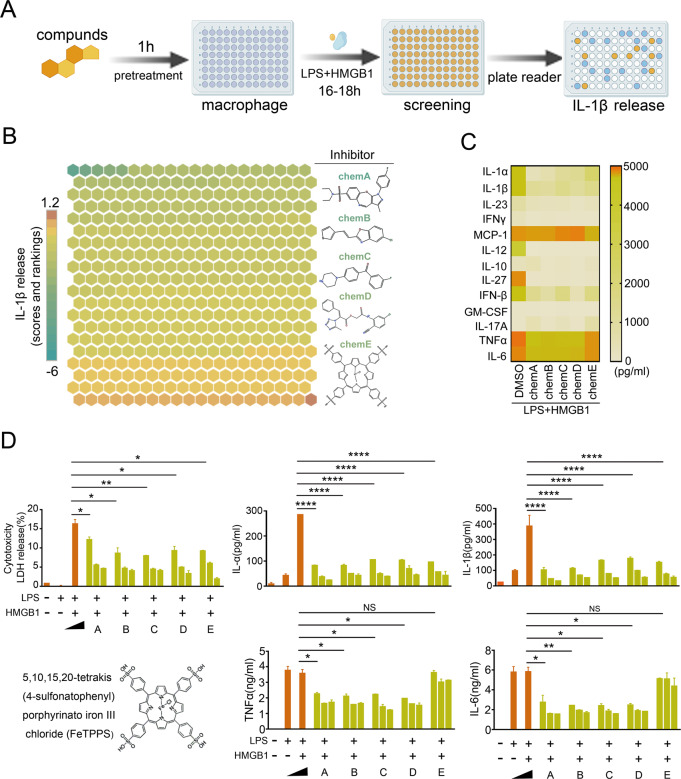
Identification of bioactive compounds inhibits the HMGB1-caspase-11 pathway. **A** A phenotypic screening strategy to identify compounds that inhibit the HMGB1-caspase-11 pathway in mouse macrophages exposed to both recombinant HMGB1 and LPS. **B** Heatmap of HMGB1-caspase-11 pathway activity changes based on IL-1β release from mouse peritoneal macrophages after stimulation with LPS (1 μg/mL) + HMGB1 (400 ng/mL) for 16 h in the absence or presence of 434 bioactive compounds (10 μM). **C** Heatmap of cytokine assay in mouse macrophages after stimulation with LPS (1 μg/mL) + HMGB1 400 ng/mL 16 h in the absence or presence of five small molecules. **D** LDH Assay and ELISA for IL-1α, IL-1β, TNFα, and IL-6 in the supernatants of mouse peritoneal macrophages after stimulation with LPS (1 μg/mL) + HMGB1 (400 ng/mL) for 16 h in the absence or presence of five small molecules with indicated dose (1, 5, and 10 μM). Graphs show the mean ± SD of technical replicates and are representative of at least three independent experiments. An unpaired *t*-test (two-sided) was used (LDH _LH vs. LH + A_ *P = 0.0494; _LH_ _vs._ _LH + B_ *P = 0.0266; _LH_ _vs._ _LH + C_ **P = 0.0095; _LH_ _vs._ _LH + D_ *P* = 0.0252; _LH_ _vs._ _LH + E_ *P = 0.0133; IL-1α, IL-1β _LH_ _vs._ _LH + A/B/C/D/E_ ****P < 0.0001; TNFα _LH_ _vs._ _LH + A_ *P = 0.0198; _LH_ _vs._ _LH + B_ *P = 0.0185; _LH_ _vs._ _LH + C_ *P = 0.0169; _LH_ _vs._ _LH + D_ *P* = 0.0119; IL-6 _LH_ _vs._ _LH + A_ *P = 0.0355; _LH_ _vs._ _LH + B_ **P = 0.0088; _LH_ _vs._ _LH + C_ *P = 0.0103; _LH_ _vs._ _LH + D_ *P* = 0.0101). Compound A: N,N-dimethyl-3-{[(2-methylquinazolin-4-yl)sul-fanyl]methylbenzene-1-sulfonamide; Compound B: 5-chloro-2-[2-(thiophen-3-yl)ethe-nyl]-1,3-benzoxazole; Compound C: (5E)-5-{[5-(4-Chlorophenyl)-2-furyl]methylene}-2thioxoimidazolidin-4 4-one; Compoud D: 2-oxo-2-(3-oxo-1,2,3,4-tetrahydroquinoxalin-1-yl) ethyl 2-[(4,4,4- trifluoro-3-oxobut-1-en-1-yl) amino] benzoate; Compound E: 5,10,15,20-Tetrakis(4-sulfonatophenyl) porphyrinato Iron (III), chloride.

We previously identified HMGB1 as a potential drug-target in caspase-11-mediated lethal immune disorders, such as sepsis^11^. In cultured mouse macrophages, recombinant HMGB1 efficiently delivers LPS into the cytoplasm, leading to caspase-11-dependent IL-1β release^11^. Taking advantage of these findings, we established a phenotypic screening strategy to identify compounds that inhibit the HMGB1-caspase-11 pathway in mouse macrophages exposed to both recombinant HMGB1 and LPS (Fig. 1A). As expected, the absence of extracellular recombinant HMGB1 or genetic deletion of Caspase-11 abolished LPS-induced IL-1β release (Fig. S1). By screening 434 compounds, we identified 12 compounds that enhanced IL-1β release. We also observed that five small molecules significantly inhibited caspase-11-dependent IL-1β release from macrophages (Fig. 1B) and also markedly inhibited caspase-11-dependent release of IL-1α and IL-1β (Fig. 1C). However, addition of compound A, B, C, and D markedly attenuated the production of TNF and interleukin-6 (IL-6) (Fig. 1D), suggesting that these compounds inhibit the global inflammatory responses. In contrast, FeTPPS exposure did not alter the production of TNF and IL-6 in macrophages (Fig. 1D). Taking together, this phenotypic screening strategy identifies FeTPPS as a potential selective inhibitor of the HMGB1-caspase-11 pathway.

### FeTPPS selectively inhibits HMGB1-mediated caspase-11 activation in vitro

**Fig. 2:**
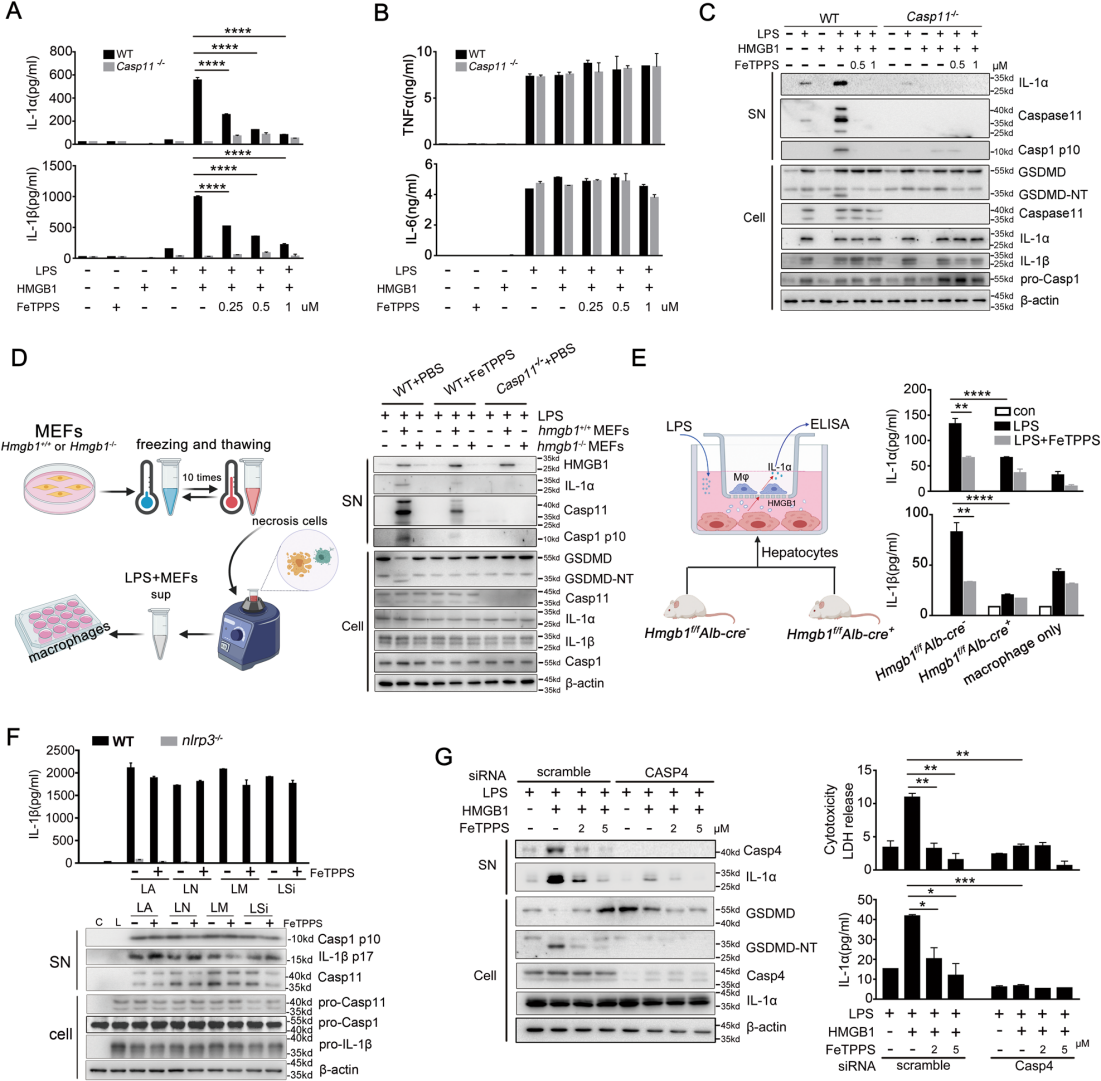
FeTPPS selectively inhibits recombinant HMGB1-mediated caspase-11 activity in vitro. **A**, **B** Production of IL-1α, IL-1β, TNFα, and IL-6 (as measured by ELISA) of WT or *Casp11*^*−/−*^ peritoneal macrophages stimulated with LPS alone (1 μg/mL) or LPS (1 μg/mL) + HMGB1 (400 ng/mL) in the absence or presence of indicated doses of FeTPPS for 16 h. Data presented as mean ± SD of technical replicates. ****P < 0.0001 (unpaired *t-test*). **C** Western blots for IL-1α, caspase-11, and caspase-1 in the supernatants or GSDMD-NT, caspase-11, IL-1α, IL-1β, caspase-1 in the cell lysates from WT or *Casp11*^*−/−*^ peritoneal macrophages stimulated with LPS alone (1 μg/mL) or LPS (1 μg/mL) + HMGB1 (400 ng/mL) in the absence or presence of indicated doses of FeTPPS for 16 h. **D** Necrotic cell lysate preparation is shown in the left panel. Western blots for HMGB1, IL-1α, caspase-11, and caspase-1 in the supernatants or GSDMD-NT, Caspase-11, IL-1α, IL-1β, and caspase-1 in the cell lysates from WT or *Casp11*^*−/−*^ peritoneal macrophages stimulated with LPS alone (1 μg/mL), LPS (1 μg/mL) + HMGB1^+/+^, or HMGB1^−/−^ MEF cells in the absence or presence of FeTPPS for 16 h. **E** Primary hepatocytes isolated from mice of indicated genotypes co-cultured with WT mouse peritoneal macrophages are shown in the left panel. IL-1a and IL-1β released from macrophages after the stimulation of LPS (1 μg/mL) in the absence or presence of FeTPPS are shown in the right panel. Data presented as mean ± SD of technical replicates. Two-way ANOVA was used (IL-1α _hmgb1f/f Albcre−_ _LPS_ _vs._ _LPS + FeTPPS_ **P = 0.004; _hmgb1f/f Albcre_−_ + LPS_ _vs._ _hmgb1f/f Albcre_^+^_ + LPS_ ****P < 0.0001; IL-1β _hmgb1f/f Albcre_^−^_LPS_ _vs._ _LPS + FeTPPS_ **P = 0.0025; _hmgb1f/f Albcre_^−^_ + LPS_ _vs._ _hmgb1f/f Albcre_^+^_ + LPS_ ****P < 0.0001). **F** Production of IL-1β (as measured by ELISA), Western blots of supernatants and cell lysates to detected IL-1β, Caspase-1 cleavage from WT or *NLRP3*^−/−^ peritoneal macrophages primed with LPS 100 ng/mL and indicated stimulation. **G** Western blots for IL-1α, caspase-4 (Casp4), GSDMD-NT in the supernatants or cell lysates. LDH assay and ELISA for IL-1α in the supernatants of PMA-primed human monocytic THP-1 cells transfected with scrambled siRNA or CASP4-specific siRNA upon HMGB1 (400 ng/mL) and LPS (1 μg/mL) stimulation in the absence or presence of FeTPPS. Data presented as mean ± SD of technical replicates. An unpaired *t*-test (two-sided) was used (LDH _LH_ _vs._ _LH + FeTPPS 2.5 μM_ **P = 0.0085; _LH_ _vs._ _LH + FeTPPS 5 μM_ **P = 0.0071; _LH scramble_ _vs._ _LH casp4 siRNA_ **P = 0.0043; IL-1α _LH_ _vs._ _LH + FeTPPS 2.5 μM_ *P = 0.0328; _LH_ _vs._ _LH + FeTPPS 5 μM_ *P = 0.0198; _LH scramble_ _vs._ _LH casp4 siRNA_ ***P = 0.004). Graphs are representative of at least three independent experiments.

To prove that FeTPPS selectively inhibits HMGB1-mediated caspase-11 activation, WT, *Caspase-11*^−/−^, *Nlrp3*^*−/−*^, or *ASC*^*−/−*^ mouse peritoneal macrophages were stimulated with recombinant HMGB1 + LPS in the presence of different concentrations of FeTPPS. We observed that FeTPPS dose-dependently inhibited the release of IL-1α and LDH, which relied on caspase-11 but not NLRP3 or ASC (Fig. 2A, Figs. S2 and S3). HMGB1 + LPS-induced IL-1β release depends on both caspase-11 and NLRP3^11^. In line with this finding, FeTPPS also inhibited the release of IL-1β (Fig. 2A, Fig. S3). By contrast, FeTPPS at the above concentrations did not alter the production of TNF or IL-6 (Fig. 2B). As revealed by western blot, addition of FeTPPS markedly attenuated the release of IL-1α and the cleavage of caspase-1 and GSDMD, a substrate of caspase-11, but failed to inhibit the expression of IL-1α, IL-1β, caspase-11, caspase-1, and GSDMD (Fig. 2C). In LPS-primed WT mouse macrophages, FeTPPS did not inhibit the release of IL-1β and the cleavage of capase-1 and IL-1β induced by ATP, MSU, nigericin and silicon crystals (Fig. 2F), all of which are well-known activators of the NACHT-, LRR-, and PYD-containing protein 3 (NLRP3) inflammasomes^16,17^.

HMGB1 released by damaged or necrotic cells is able to elicit caspase-11-dependent immune responses in the presence of LPS^11^. To confirm that FeTPPS could selectively inhibit HMGB1- and LPS-mediated caspase-11 activation, we stimulated WT or caspase-11-deficient mouse macrophages with LPS and lysates of necrotic *Hmgb1*^+/+^ or *Hmgb1*^−/−^ MEFs. Addition of FeTPPS markedly reduced the release of IL-1α and the cleavage of caspase-1 in the supernatants (Fig. 2D). FeTPPS exposure did not alter the production of TNF or IL-6 (Fig. S4). As hepatocyte-released HMGB1 is critical for caspase-11-mediated immune responses in endotoxemia or sepsis^11^, we next tested whether FeTPPS could inhibit hepatocyte HMGB1-mediated caspase-11 activation. *Hmgb1*^+/+^ or *Hmgb1*^−/−^ primary mouse hepatocytes were co-cultured with mouse peritoneal macrophages before LPS stimulation (Fig. 2E). As expected, LPS only induced IL-1α and IL-1β release from WT mouse macrophages co-cultured with *Hmgb1*^+/+^ hepatocytes. These caspase-11-dependent immune responses were abrogated by FeTPPS (Fig. 2E). Caspases-4 is one of the human homologs of Caspase-11. Next, we measured whether FeTPPS could inhibit HMGB1-caspase-4 pathway in human cells. Silencing of Caspase-4 expression blocked HMGB1-induced IL-1a and LDH release from human monocytic THP-1 cells in the presence of LPS (Fig. 2G). These caspase-4-dependent immune responses were abrogated by FeTPPS (Fig. 2G). Taken together, these observations demonstrated that FeTPPS selectively inhibits HMGB1-mediated caspase-11/caspase-4 activation in vitro.

### FeTPPS attenuates caspase-11–mediated immune responses and lethality in endotoxemia and bacterial sepsis

**Fig. 3:**
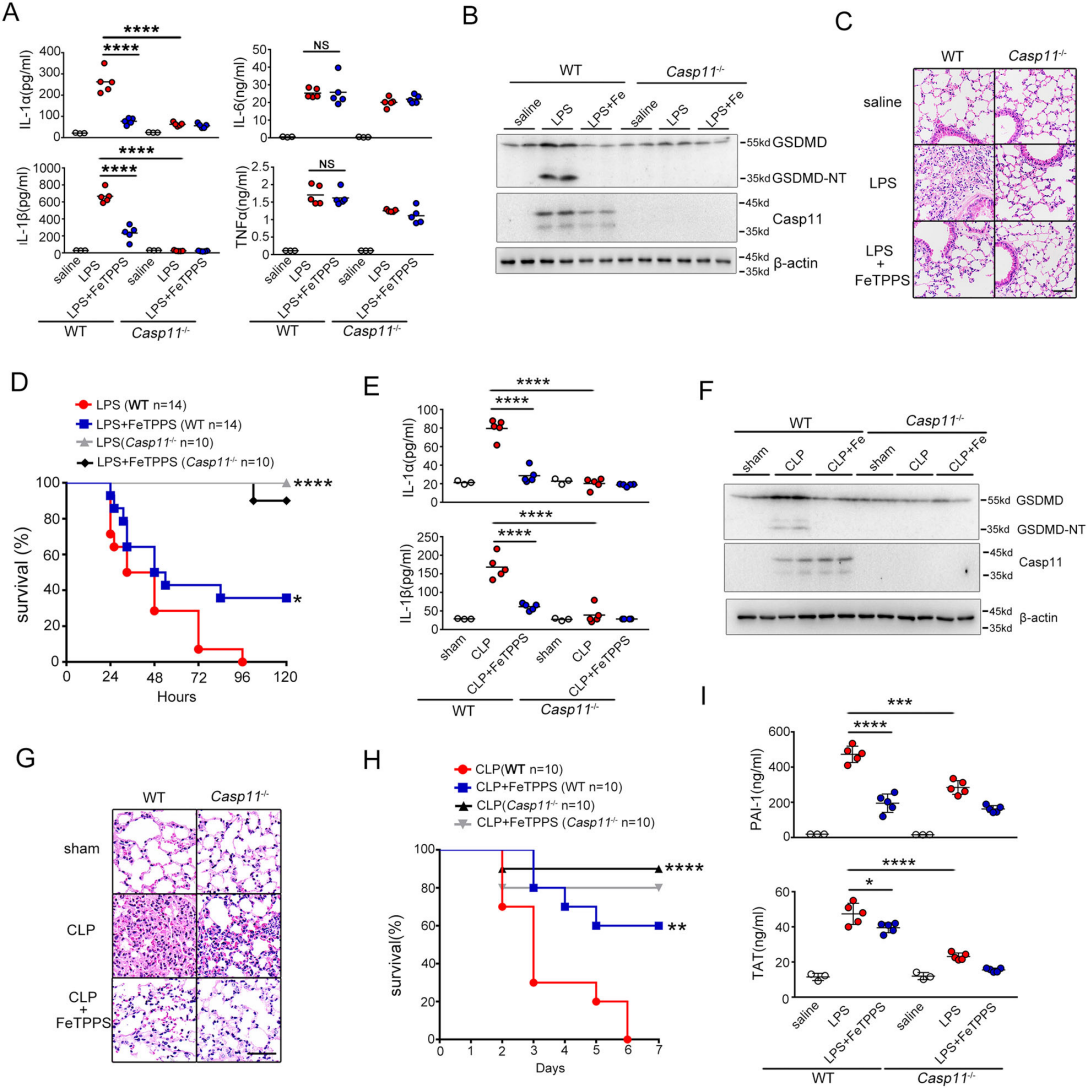
FeTPPS attenuated caspase-11-dependent immune responses and lethality in sepsis. **A–D** WT or *Casp11*^*−/*^^−^ mice were pretreated with FeTPPS 6 mg/kg or saline by intraperitoneal injection 1 h before intraperitoneal challenge with 25 mg/kg LPS. Serum levels of IL-1α, IL-1β, TNFα, IL-6 (**A**) (unpaired *t*-test, *n* = 5, ****P < 0.0001), western blots for GSDMD-NT, caspase-11 in lung (**B**), hematoxylin and eosin (H&E) stained sections of lung. Scale bar: 50 μm (**C**) and kaplan–Meier survival curves (**D**). Significance assessed using Log-rank test: LPS (WT *n* = 14) vs. LPS + FeTPPS (WT *n* = 14), *P = 0.0422; WT LPS (*n* = 14) vs. Casp11^−/−^ LPS (*n* = 10), ****P < 0.0001. **E**–**H** WT or *Casp11*^*−/−*^ mice were pretreated with FeTPPS 6 mg/kg or saline by intraperitoneal injection 1 h before subjected to cecum ligation and puncture (CLP) or sham operation. Serum levels of IL-1α, IL-1β (**E**), western blots for GSDMD-NT,Caspase-11 in lung (**F**), hematoxylin and eosin (H&E) stained sections of lung. Scale bar: 50 μm (**G**) and kaplan–Meier survival curves (**H**). Significance assessed using Log-rank test: CLP (WT *n* = 10) vs. CLP + FeTPPS (WT *n* = 10), **P = 0.0036; CLP WT (*n* = 10) vs. Casp11^−^^/−^ (*n* = 10), ****P < 0.0001. **I** WT or *Casp11*^*−/−*^ mice were injected with FeTPPS 6 mg/kg 1 h before intraperitoneal challenge with 25 mg/kg LPS. Plasma concentrations of PAI-1 and TAT were measured. An unpaired *t*-test was used in PAI-1 _LPS(WT *n* = 5)_ _vs._ _LPS+FeTPPS (WT *n* = 5)_, ****P < 0.0001; _WT LPS (*n* = 5)_ _vs._ _Casp11_^−/−^_LPS (*n* = 5)_ ***P = 0.0001 and TAT _LPS (WT *n* = 5)_ _vs._ _LPS + FeTPPS (WT *n* = 5)_, *P < 0.0299; _WT LPS (*n* = 5)_ _vs._ _Casp11_^−/−^_LPS (*n* = 5)_ ****P < 0.0001.

Next, we sought to determine whether FeTPPS could attenuate caspase-11-mediated immune responses in vivo. Caspase-11-deficient and WT mice injected with lethal dose of LPS concomitant with or without FeTPPS treatment. Caspase-11-dependent release of IL-1α and IL-1β was markedly inhibited by FeTPPS administration while did not affect the release of TNF or IL-6 (Fig. 3A). Consistent with previous findings^18^, ASC deficiency did not significantly reduce the IL-1α release and the lethality in endotoxemia (Fig. S5). Administration of FeTPPS significantly improved the survival and markedly reduced the IL-1α release in both WT and ASC^−/−^ mice (Fig. S5). These results further support the notion that FeTPPS selectively inhibits caspase-11-dependent immune responses. To directly test whether FeTPPS could inhibit caspase-11 activation in vivo, we measured the enzymatic cleavage of GSDMD, the substrate of caspase-11, in the intestine tissues. As revealed by western blot, deletion of caspase-11 abolished the GSDMD cleavage in endotoxemia (Fig. 3B). Administration of FeTPPS prevented endotoxemia-induced GSDMD cleavage in a manner similar to caspase-11 deficiency (Fig. 3B). Accordingly, FeTPPS treatment markedly attenuated caspase-11-dependent lung injury and significantly promoted survival in lethal endotoxemia (Fig. 3C, D).

To further confirm that FeTPPS inhibits caspase-11 activation in vivo, experiments were then conducted to determine whether FeTPPS treatment could attenuate caspase-11-dependent immune responses and lethality in bacterial sepsis. Using CLP, a clinically relevant murine model of gram-negative polymicrobial sepsis, we observed that FeTPPS treatment markedly inhibited caspase-11-dependent release of IL-1α and IL-1β, but did not affect the secretion of TNF or IL-6 (Fig. 3E, Fig. S6). Further, administration of FeTPPS prevented caspase-11-mediated GSDMD cleavage in the intestine tissues, markedly attenuated caspase-11-dependent lung injury, significantly promoted survival after CLP (Fig. 3F–H) and reduced caspase-11-dependent secretion of TAT and PAI-1 in plasma of endotoxemic mice (Fig. 3I). Taken together, these observations indicated that FeTPPS attenuates caspase-11 activation in vivo, which is driven by hepatocyte-released HMGB1 in endotoxemia or bacterial sepsis.

### FeTPPS inhibits HMGB1-mediated cytosolic delivery of LPS

**Fig. 4:**
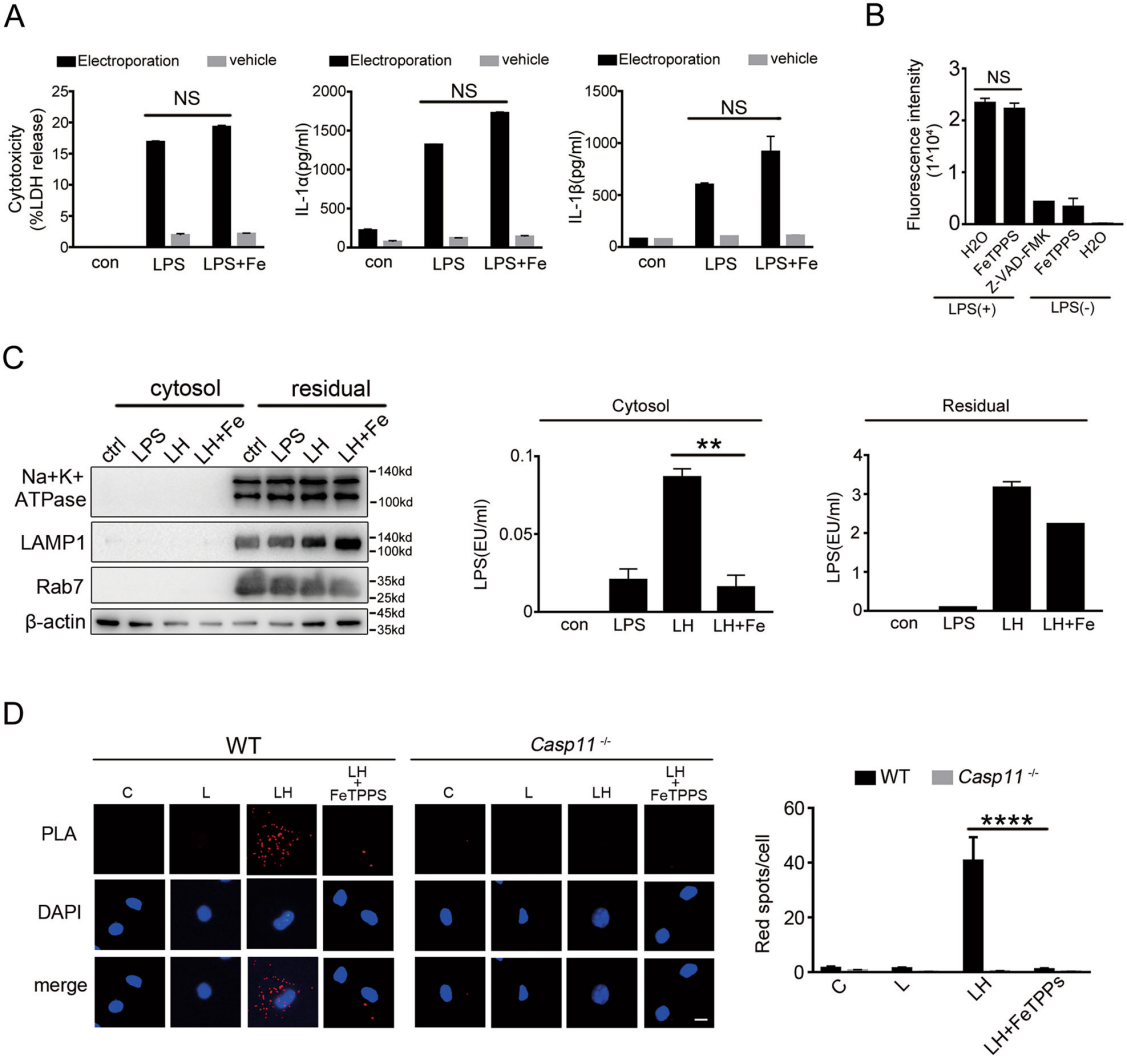
FeTPPS inhibits HMGB1-mediated cytosolic delivery of LPS. **A** LDH and IL-1α, IL-1β release 16 h post-LPS transfection with or without FeTPPS from WT or *Casp11*^*−/−*^ peritoneal macrophages. **B** LPS-induced activation of insect cell-derived caspase-11 in the presence or the absence of FeTPPS or pan-caspase inhibitor zVAD-FMK. H_2_O was added as control. Caspase activity was determined by measuring the fluorescence intensity of free AMC hydrolyzed from zVAD-AMC. **C** Western blots for Na+/K+ ATPase, LAMP1, Rab7 (left panel), and LAL assay for LPS (EU, endotoxin units) in the cytosolic and residual fractions of mouse peritoneal macrophages treated with LPS alone (1 μg/mL) or LPS (1 μg/mL) + HMGB1 (400 ng/mL) in the absence or presence of FeTPPS for 2 h. Data presented as mean ± SD of technical replicates. An unpaired *t*-test (two-sided) was used. **P = 0.0089. **D** Interaction between caspase-11 and LPS was visualized as red spots under fluorescence microscopy using the proximity-ligation assay (PLA). Mouse peritoneal macrophages stimulated with LPS alone (5 μg/mL) or LPS (5 μg/mL) + HMGB1 (10 μg/mL) (LH) in the absence or presence of FeTPPS (1 μM) for 2 h. Scale bar: 10 μm. Data presented as mean ± SD of technical replicates. An unpaired *t*-test (two-sided) was used. ****P < 0.0001. Graphs are representative of at least three independent experiments.

Next, we investigated the mechanisms of how FeTPPS inhibits HMGB1- and LPS-induced caspase-11 activation. When we artificially delivered LPS directly to the cytosol of mouse macrophages by electroporation, we found that addition of FeTPPS failed to inhibit caspase-11-dependent release of IL-1α and IL-1β (Fig. 4A). To provide further evidence that FeTPPS does not directly inhibit the enzymatic activity of caspase-11, we used bioactive recombinant caspase-11 in a cell-free system, in which the activity of caspase-11 can be measured using zVAD-AMC. Addition of FeTPPS failed to inhibit LPS-induced caspase-11 activation (Fig. 4B). By contrast, zVAD-FMK, a pan-caspase inhibitor, markedly reduced caspase-11 activity (Fig. 4B). These observations clearly suggested that FeTPPS is not a direct inhibitor of caspase-11. HMGB1-mediated cytosolic delivery of LPS is critical for caspase-11 activation in endotoxemia or sepsis. We next tested whether FeTPPS inhibits this process and used low concentration of digitonin to isolate the cytosol fraction devoid of cytoplasmic membranes, endosomes or lysosomes from mouse macrophages that were stimulated with LPS and HMGB1 (Fig. 4C). As revealed by LPS activity assay, addition of FeTPPS markedly inhibited HMGB1-mediated cytosolic delivery of LPS (Fig. 4C, Fig. S7). To confirm this phenomenon, we examined the physical interaction between LPS and caspase-11 by using a PLA. Consistent with our previous observations, recombinant HMGB1 significantly enhanced the LPS-caspase-11 interaction (Fig. 4D), manifested by the cytosolic co-localization of LPS with caspase-11. Addition of FeTPPS to the cell-culture medium diminished the detection of LPS-caspase-11 interaction in the cytosol (Fig. 4D). Together, these findings indicate that FeTPPS prevents caspase-11 activation by inhibiting HMGB1-mediated cytosolic delivery of LPS.

### FeTPPS disrupts the HMGB1-LPS binding

**Fig. 5:**
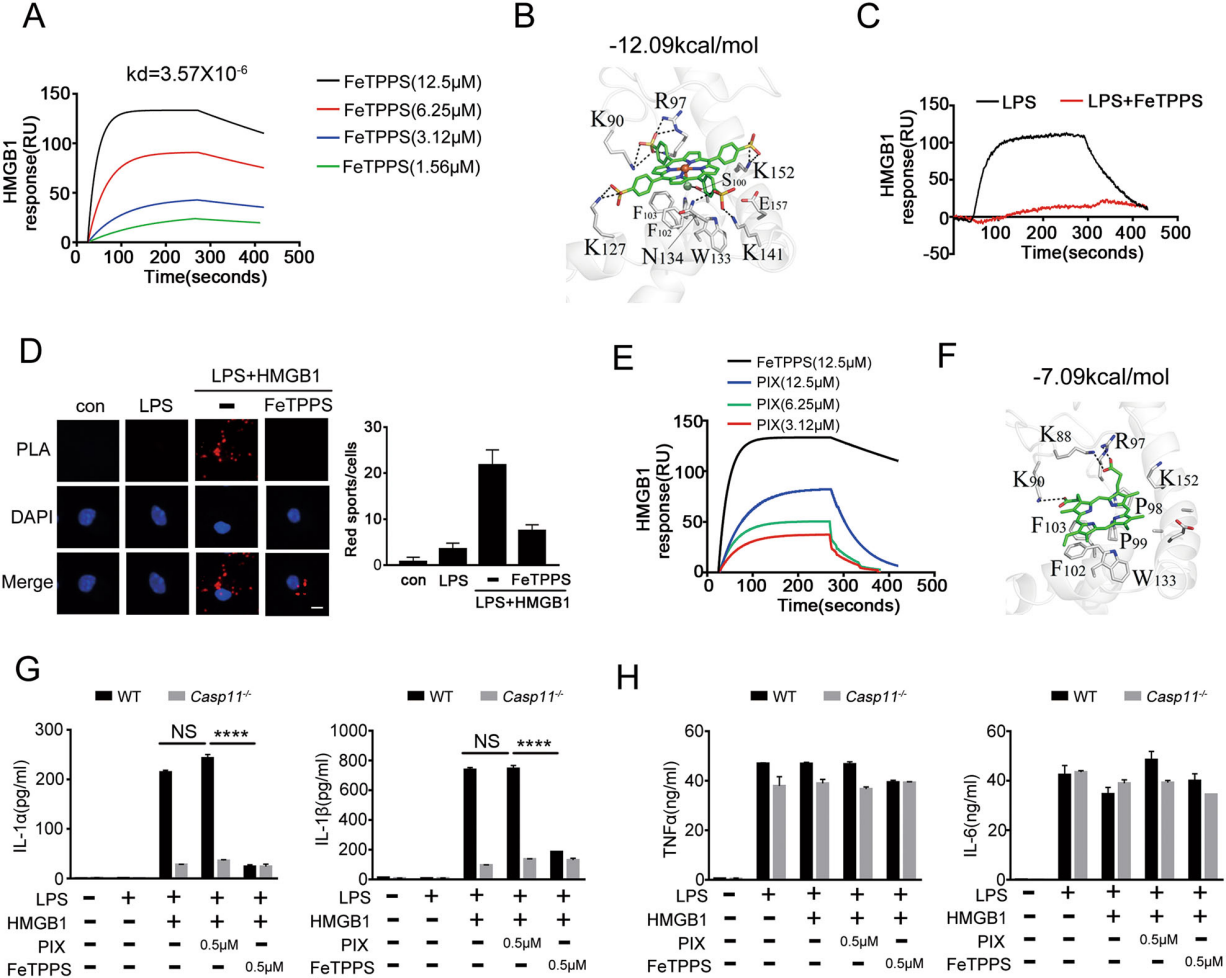
FeTPPS disrupts the HMGB1-LPS binding. **A** The sensorgrams of FeTPPS (1.56, 3.12, 6.25, 12.5 μM) binding to the HMGB1 chip-immobilized proteins are expressed in RU (response unit) vs. time after subtracting the control signal. **B** The 3D binding model between FeTPPS and HMGB1. The protein is shown as cartoon, the compound is colored in green. High-scoring docking poses of FeTPPS and HMGB1 was −12.09 kcal/mol. Ionic bonds are shown as dash lines. **C** The sensorgrams of LPS binding to the HMGB1 chip-immobilized proteins with or without FeTPPS are expressed in RU (response unit) vs. time after subtracting the control signal. **D** Interaction between HMGB1 and LPS was visualized as red spots under fluorescence microscopy using the proximity-ligation assay (PLA). Mouse peritoneal macrophages stimulated with LPS alone (5 μg/mL) or LPS (5 μg/mL) + HMGB1 (10 μg/mL) (LH) in the absence or presence of FeTPPS (1 μM) for 2 h. Scale bar: 10 μm. **E** The binding affinity of FeTPPS or Protoporphyrin IX (PIX) with recombinant HMGB1 (immobilized) are expressed in RU (response unit) vs. time after subtracting the control signal. **F** The 3D binding model between PIX and HMGB1. The protein is shown as cartoon, the compound is colored in green. High-scoring docking poses of PIX and HMGB1 was −7.09 kcal/mol. Ionic bonds are shown as dash lines. **G**, **H** Production of IL-1α, IL-1β, TNFα and IL-6 (as measured by ELISA) from WT or *Casp11*^*−/−*^ peritoneal macrophages stimulated with LPS (1 μg/mL) + HMGB1 (400 ng/mL) in the absence or presence of PIX or FeTPPS for 16 h. Data presented as mean ± SD of technical replicates. An unpaired *t*-test (two-sided) was used. ****P < 0.0001. Graphs are representative of at least three independent experiments.

Next, we investigated the mechanisms by which FeTPPS inhibits HMGB1-mediated cytosolic delivery of LPS. By using surface plasmon resonance, we found that FeTPPS could bind HMGB1 with a high affinity (Fig. 5A). MOE^1^ Dock was used for molecular docking between ligands and receptor and predicting the binding affinity. FeTPPS and HMGB1 were defined as the ligand and the receptor, respectively, the docking score of the FeTPPS-HMGB1 binding affinity was −12.09 kcal/mol. This computational analysis also revealed that four sulfonic groups on FeTPPS formed ionic bonds with the amino acid residues (K90, R97, K127, K141, and K152) of HMGB1. Particularly one of the sulfonic groups on FeTPPS formed hydrogen bond with the residue (N134) of HMGB1. The chloridion from the Fe–Cl formed a hydrogen bond with the residue (S100) of HMGB1 (Fig. 5B). Together with our previous findings that HMGB1-LPS binding is essential for HMGB1-mediated cytosolic delivery of LPS, we reasoned that FeTPPS might inhibit the physical interaction between HMGB1 and LPS. Indeed, FeTPPS disrupted the HMGB1-LPS binding (Fig. 5C). To support this notion, we assessed the HMGB1-LPS interactions by using PLA and observed that the addition of FeTPPS markedly reduced the HMGB1-LPS interactions (Fig. 5D).

To provide further evidence that FeTPPS inhibits HMGB1-mediated caspase-11 activation through the disruption of HMGB1-LPS binding, we conducted experiments with PIX, a small molecule whose structure is similar to that of FeTPPS but lacks of sulfonic groups. The docking score of the PIX-HMGB1 binding affinity was **−**7.09 kcal/mol revealed the binding affinity between PIX and HMGB1 was much lower than that of FeTPPS-HMGB1 interaction, due to the loss of salt bridges and hydrogen bonds (Fig. 5F). Surface plasmon resonance confirmed that PIX bound HMGB1 with a much lower affinity as compared to the FeTPPS-HMGB1 interaction (Fig. 5E). In contrast to FeTPPS, PIX failed to inhibit caspase-11-dependent release of IL-1α and IL-1β from mouse macrophages stimulated with HMGB1 and LPS (Fig. 5G). Both FeTPPS and PIX did not alter the secretion of TNF and IL-6 (Fig. 5H). Collectively, these data establish that FeTPPS inhibits HMGB1-mediated caspase-11 activation, at least in part, through disruption of the HMGB1-LPS binding.

### FeTPPS decreases the capacity of HMGB1 to induce lysosomal rupture

**Fig. 6:**
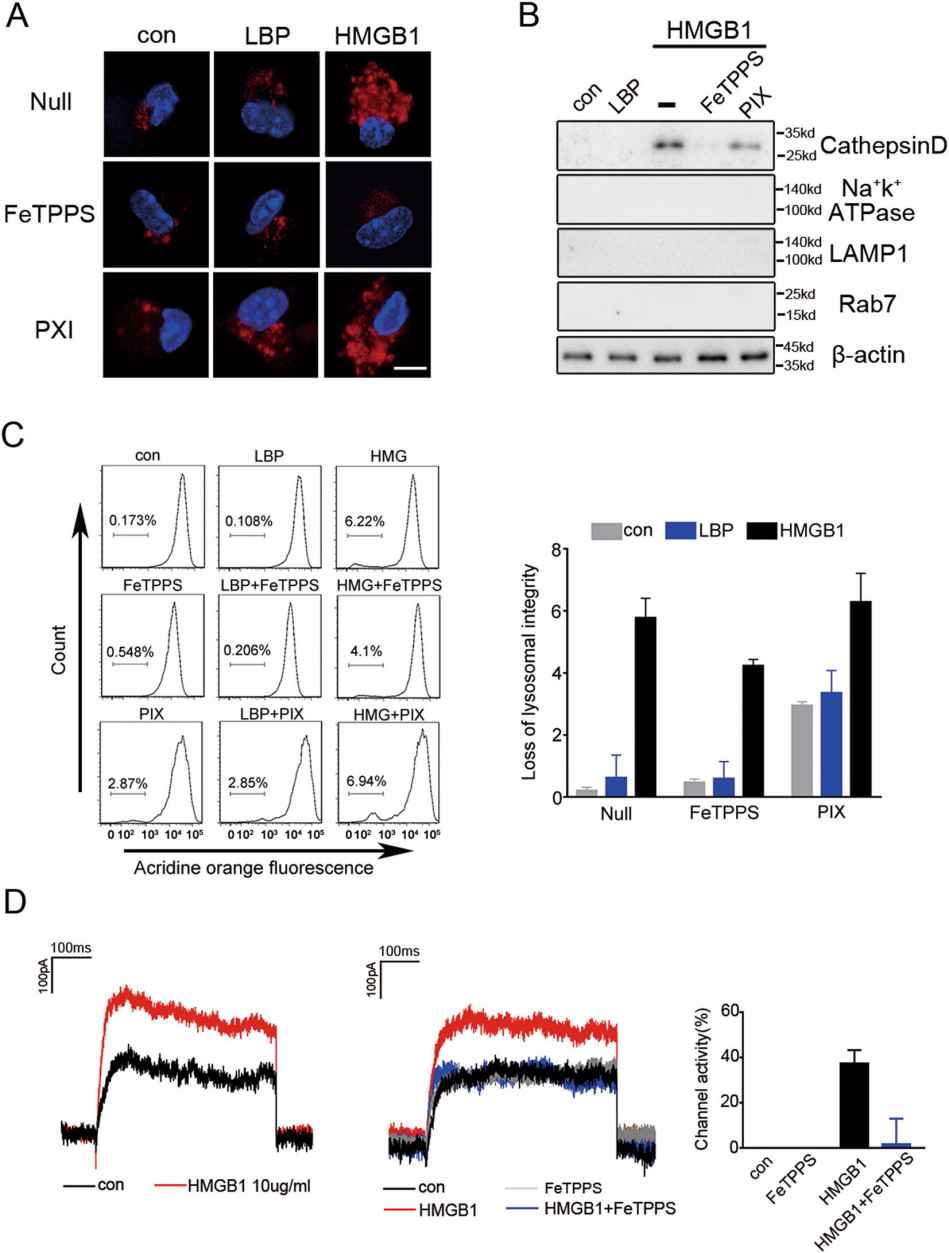
FeTPPS inhibits the cytosolic delivery of LPS through preventing HMGB1-induced lysosomal rupture. **A** Confocal microscopy of mouse peritoneal macrophages incubated for 4 h with DQ ovalbumin (10 μg/mL; red) alone or together with LPS-binding protein (10 μg/mL), or together with HMGB1(10 μg/mL) in the absence or presence of FeTPPS (1 μM) or PIX (1 μM) for 4 h, then stained with DAPI (blue). Scale bar: 10 μm. **B** Western blots for cathepsin D, Na^+^-K^+^-ATPase, Lamp1, Rab7 in the cytosolic fraction from vehicle-treated or LBP (5 μg/mL) or HMGB1 (5 μg/mL) with or without FeTPPS(1 μM) or PIX (1 μM)-treated mouse peritoneal macrophages. **C** Flow cytometry of mouse peritoneal macrophages stained with acridine orange and then treated for 4 h with LBP (10 μg/mL) or HMGB1 (10 μg/mL) with or without FeTPPS (1 μM) Numbers above bracketed lines indicate percent cells with loss of lysosomal staining with acridine orange (excitation, 488 nm; emission, 650–690 nm). **D** Whole-cell patch-clamp recording of HMGB1 in the absence or presence of FeTPPS (1 μM) induced inward current across the cytoplasmic membrane in proximity to the patch-clamp of HEK293 cells at acidic conditions (pH = 5.0). Graphs show the mean ± SD of technical replicates and are representative of at least three independent experiments.

We previously showed that the permeabilization of lysosomal membranes by HMGB1 is also a critical step for HMGB1-mediated cytosolic delivery of LPS. In this context, HMGB1 inside lysosomes permeabilizes membranes, a process that is more efficient in the acidic environment of the lysosomes. This event leads to the leakage of LPS into the cytosol and the subsequent caspase-11 activation. As FeTPPS directly binds HMGB1, we next investigated whether FeTPPS is capable of inhibiting HMGB1-induced lysosomal rupture. Consistent with our previous findings^11^, exposure to HMGB1 but not LBP, a hepatocyte-secreted LPS chaperon protein, led to the transfer of dextran from the phagosomes to the cytosol (Fig. 6A). Addition of FeTPPS but not PIX to cell-culture medium reduced the leakage of dextran to the cytosol (Fig. 6A). To confirm that FeTPPS inhibits HMGB1-induced permeabilization of lysosomal membranes with release of lysosomal contents into the cytosol, we isolated the cytosolic fraction of macrophages and measured the amounts of cathepsin D, a lysosomal protease. Notably, exposure to HMGB1 but not LBP resulted in the increase of cathepsin D in the cytoplasmic compartment, which was markedly inhibited by FeTPPS but not PIX (Fig. 6B). Next, we used flow cytometry with acridine orange staining to quantitatively measure lysosomal integrity. As expected, exposure to HMGB1 but not LBP resulted in a disappearance of lysosomes, which was significantly inhibited by FeTPPS but not PIX (Fig. 6C). To determine that FeTPPS inhibits HMGB1-induced membrane permeabilization at the acidic environment, we utilized the whole-cell patch-clamp technique, which is able to detect the inward current induced by cell membrane permeabilization^19^. As expected, HMGB1 exposure rapidly induced an inward current across cell membranes (Fig. 6D). Addition of FeTPPS significantly reduced HMGB1-induced inward current across cell membranes (Fig. 6D). Together with other data, these findings demonstrate that FeTPPS inhibits HMGB1-mediated cytosolic delivery of LPS and attenuates caspase-11-dependent immune responses, at least in part, through the inhibition of both the HMGB1-LPS binding and the HMGB1-induced lysosomal damage.

## Discussion

We previously showed that HMGB1-mediated caspase-11 activation is critical for the development of DIC, the multiple organ injury and the lethality in sepsis^9,11^. In gram-negative sepsis, hepatocytes release HMGB1 into the bloodstream after TLR4 activation by circulating LPS or gram-negative bacteria^11^. Extracellular HMGB1, in turn, binds and delivers LPS into the cytoplasm of endothelial cells or macrophages, leading to the enzymatic cleavage of GSDMD by caspase-11^9,11,12^. Excessive GSDMD activation could trigger the systemic activation of coagulation cascades, culminating in DIC, multiple organ failure, and lethality^9^. Though TLR4 signaling is required for HMGB1 release and caspase-11 expression in gram-negative sepsis, targeting the TLR4 pathway fails to improve the outcome of sepsis in clinical trial^3^. This could be partially explained by previous findings showing that TLR4 is essential for bacterial clearance and survival in bacterial sepsis^20^. TLR4-mediated cytokine (e.g., TNF) production is important for anti-microbial immune responses^20^. Consistently, a clinical study shows that neutralizing TNF by monoclonal antibody even promotes the mortality in septic patients^4^. These observations clearly suggest that selectively inhibition of deleterious host responses, such as HMGB1-caspase-11 pathway-mediated inflammation and coagulopathy^9,11,12^, rather than suppression of the global immune responses might improve the outcome of septic patients. In current study, we discovered that FeTPPS, a small molecule, could selectively inhibit HMGB1-mediated caspase-11 activation. FeTPPS treatment prevented caspase-11-dependent lethality in endotoxemia and bacterial sepsis.

At the molecular level, FeTPPS inhibits the HMGB1-LPS interaction and the HMGB1-induced lysosomal membrane permeabilization, both of which are critical for HMGB1-mediated cytosolic delivery of LPS and subsequent caspase-11 activation^11^. FeTPPS is a highly active ONOO^-^ decomposition catalyst with antioxidative activity due to the water-soluble iron (III) located in the center of porphyrin structure^21^. Administration of FeTPPS could attenuated ischemia/reperfusion injury in multiple organs^22^. However, we found that Fe(III) is dispensable for its capacity to inhibit HMGB1-mediated caspase-11 activation, suggesting that FeTPPS suppresses HMGB1-mediated cytosolic delivery of LPS independent of antioxidative activity (Fig. S8). In line with this findings, addition of NAC, a well-known antioxidant, MEG, a peroxynitrite scavenger, or iNOS inhibitors, such as dihydrochloride (1400W), L-NAME, and SMT, all of which failed to inhibit caspase-11 activation induced by HMGB1 and LPS (Fig. S9). By contrast, the four sulfonic groups on the porphyrin structure are essential for FeTPPS’ inhibitory property on HMGB1-mediated caspase-11 activation. These sulfonic groups are predicted to form salt bridges or hydrogen bonds with the amino acid residues (K90, R97, K127, N134, K141, and K152) of HMGB1. It is conceivable that the physical HMGB1-FeTPPS interaction could disrupt the binding between HMGB1 and polar lipids, such as LPS or phosphatidylserine on the lysosomal membranes, which merits further investigations.

It is noteworthy that LPS is not the only partner of HMGB1 during infection or sterile injury. Instead, HMGB1 is capable of binding various host- or microbe-derived molecules and altering their biological functions^23^. Early works show that HMGB1 is an abundant non-histone chromatin-binding protein that changes the DNA helical structure^24^. The physical interaction between HMGB1 and DNA not only shapes the structure of chromatin and protects the DNA from oxidative stress, but also enhances the immunogenic activity of nucleic acids^23,25–28^. Interestingly, HMGB1 could promote DNA- or RNA-induced host responses both outside and inside the cells. Extracellular HMGB1 binds and augments CpG DNA- or nucleosomes-induced innate immune responses through its cell-surface receptors, such as the RAGE and TLR2^26–28^, while intracellular HMGB1 facilitates DNA recognition by cyclic GMP-AMP synthase and increases the production of type 1 interferons^29^. Extracellular HMGB1 could also bind IL-1 family cytokines and increase their proinflammatory activity^30^. Further, bacterial lipids (e.g., lipid IVa and lipid A) form a complex with HMGB1, and that this complex triggers receptor-interacting protein kinase 3- and TIR domain-containing adapter-inducing IFN-β-dependent programmed cell death and inflammation^31^. Therefore, it is not surprising that HMGB1 participates in a variety of inflammatory disorders, such as sepsis, pancreatitis, rheumatoid arthritis, and systemic lupus erythematosus^32^. As the physical interaction between HMGB1 and those molecules is crucial for the augmented immune responses, these findings raise an intriguing possibility that FeTPPS might broadly disrupt the interaction between HMGB1 and its binding partners, and are able to attenuate many HMGB1-driven diseases. Taken together, current study sheds light on the development of HMGB1-targeting therapeutics and might open a new avenue to treat many immune disorders, such as sepsis.

## Supplementary information

FigS1

FigS2

FigS3

FigS4

FigS5

FigS6

FigS7

FigS8

FigS9

FigS10

FigS11

Supplementary informations
