# Supplementary figures and images for: A small molecule binding HMGB1 inhibits caspase-11-mediated lethality in sepsis
Source: Cell Death Dis. 2021 Apr 14;12(4):402. doi: 10.1038/s41419-021-03652-5 (PMC8047024; doi:10.1038/s41419-021-03652-5)

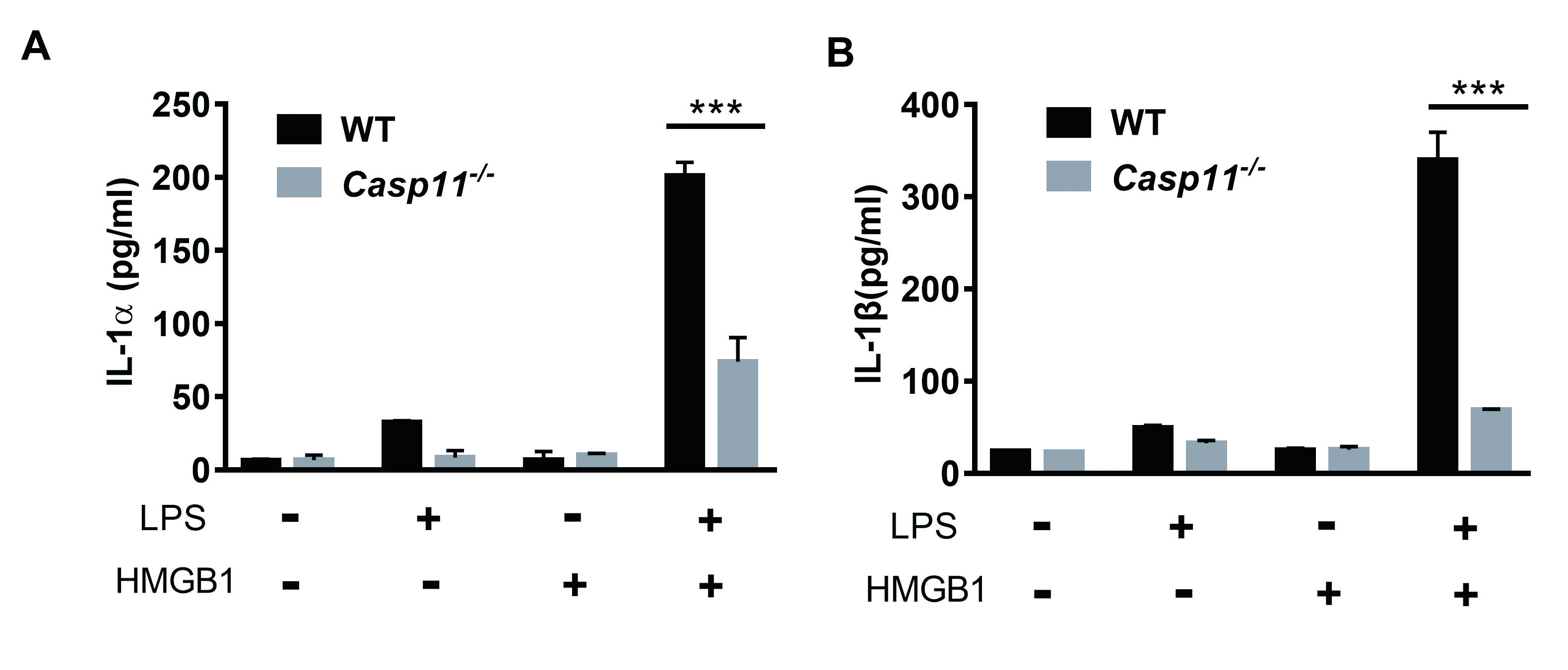

Supplement: Supplementary file 1 — FigS1 [file 41419_2021_3652_MOESM1_ESM.tif]

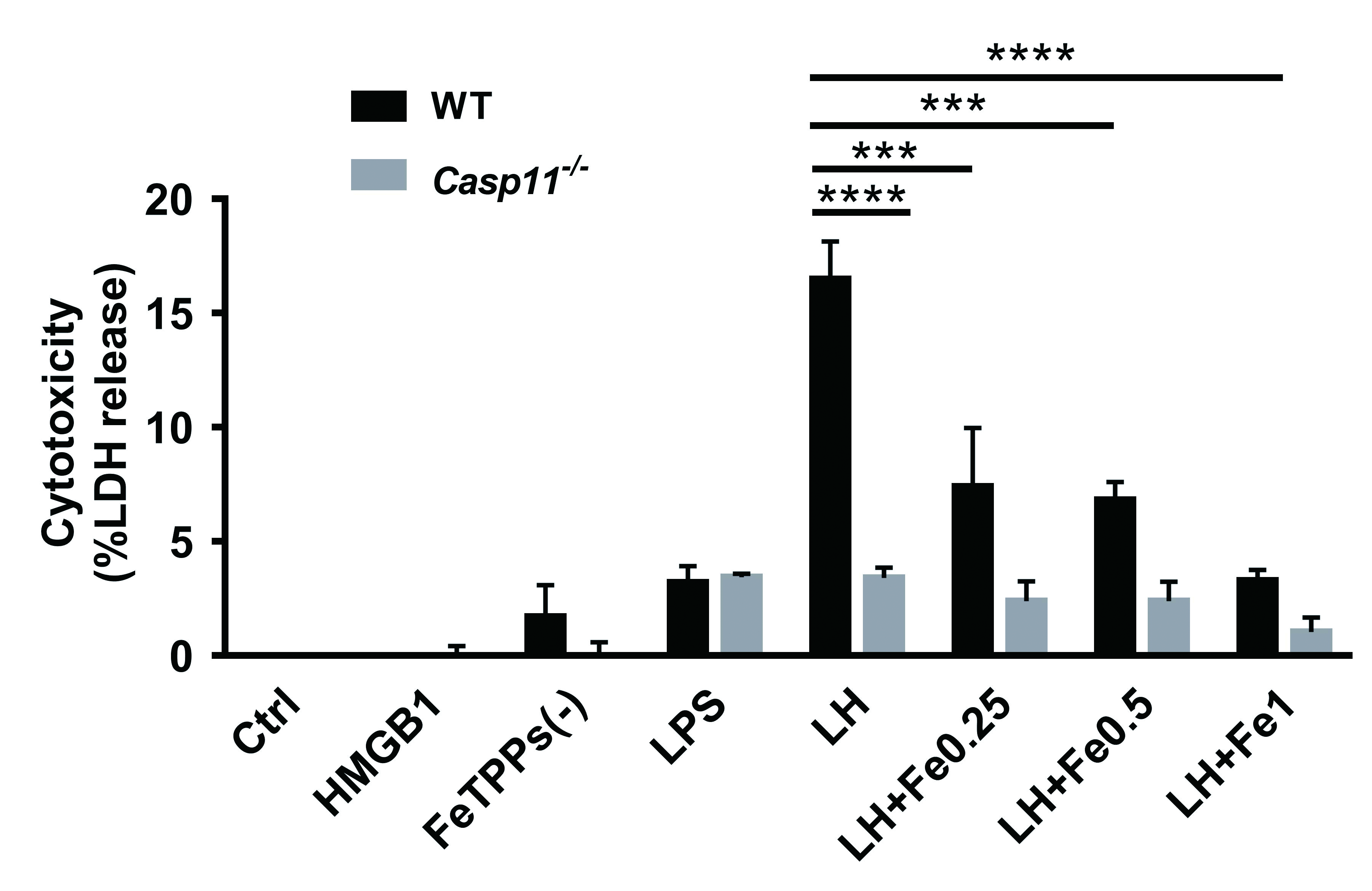

Supplement: Supplementary file 2 — FigS2 [file 41419_2021_3652_MOESM2_ESM.tif]

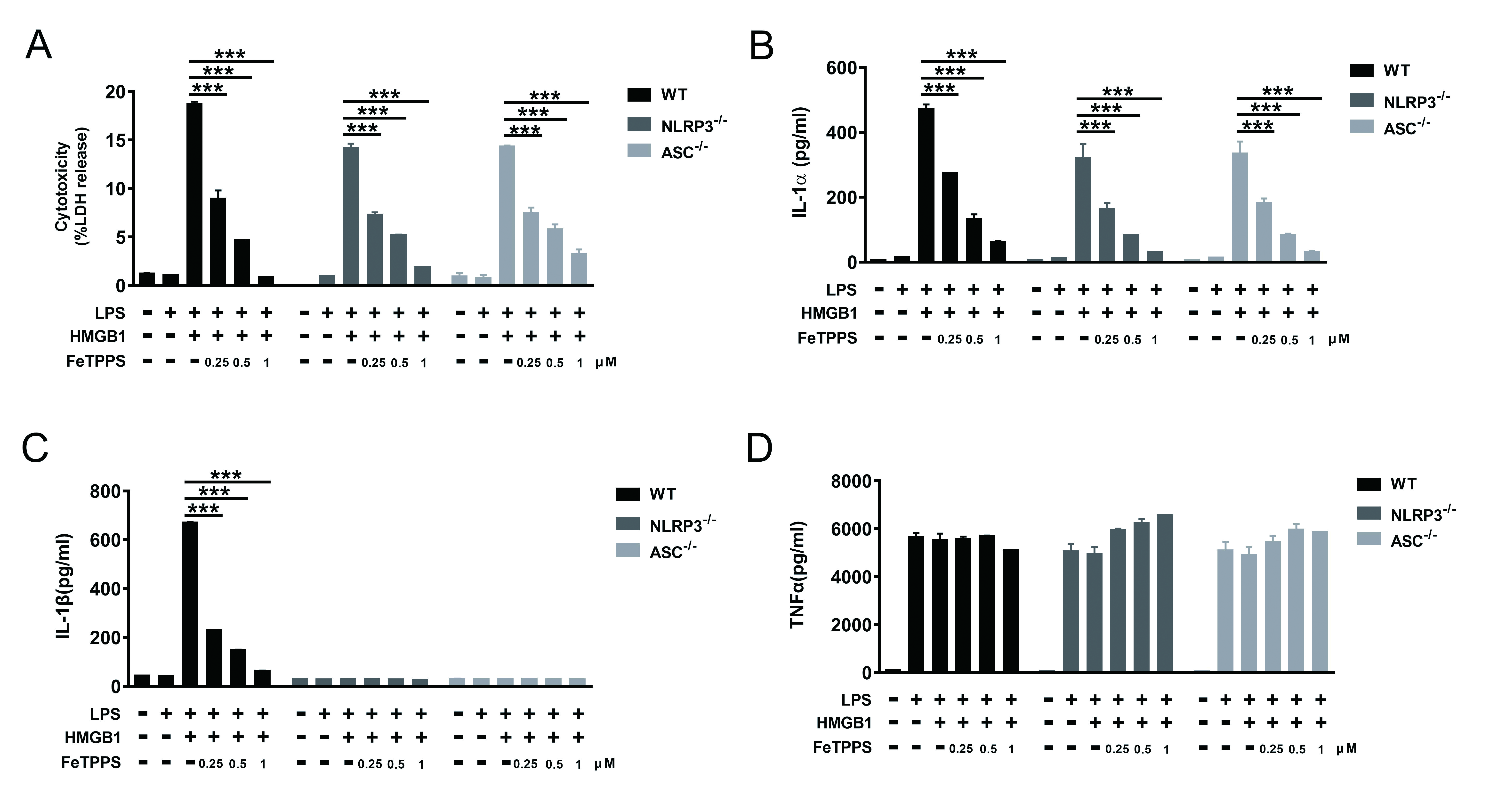

Supplement: Supplementary file 3 — FigS3 [file 41419_2021_3652_MOESM3_ESM.tif]

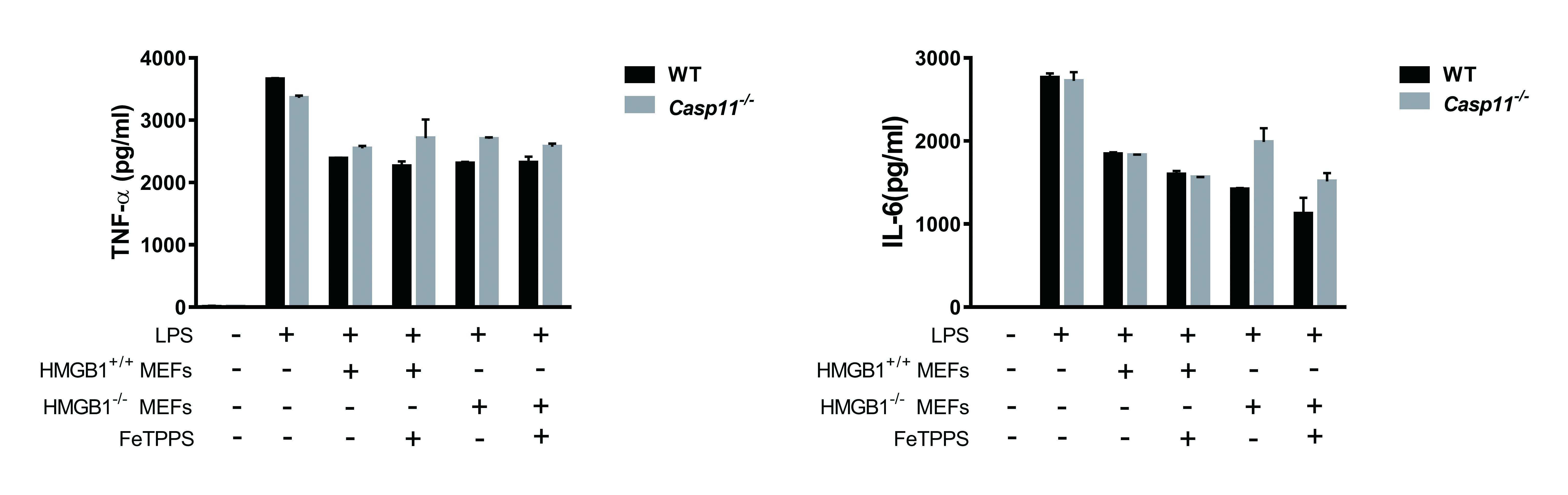

Supplement: Supplementary file 4 — FigS4 [file 41419_2021_3652_MOESM4_ESM.tif]

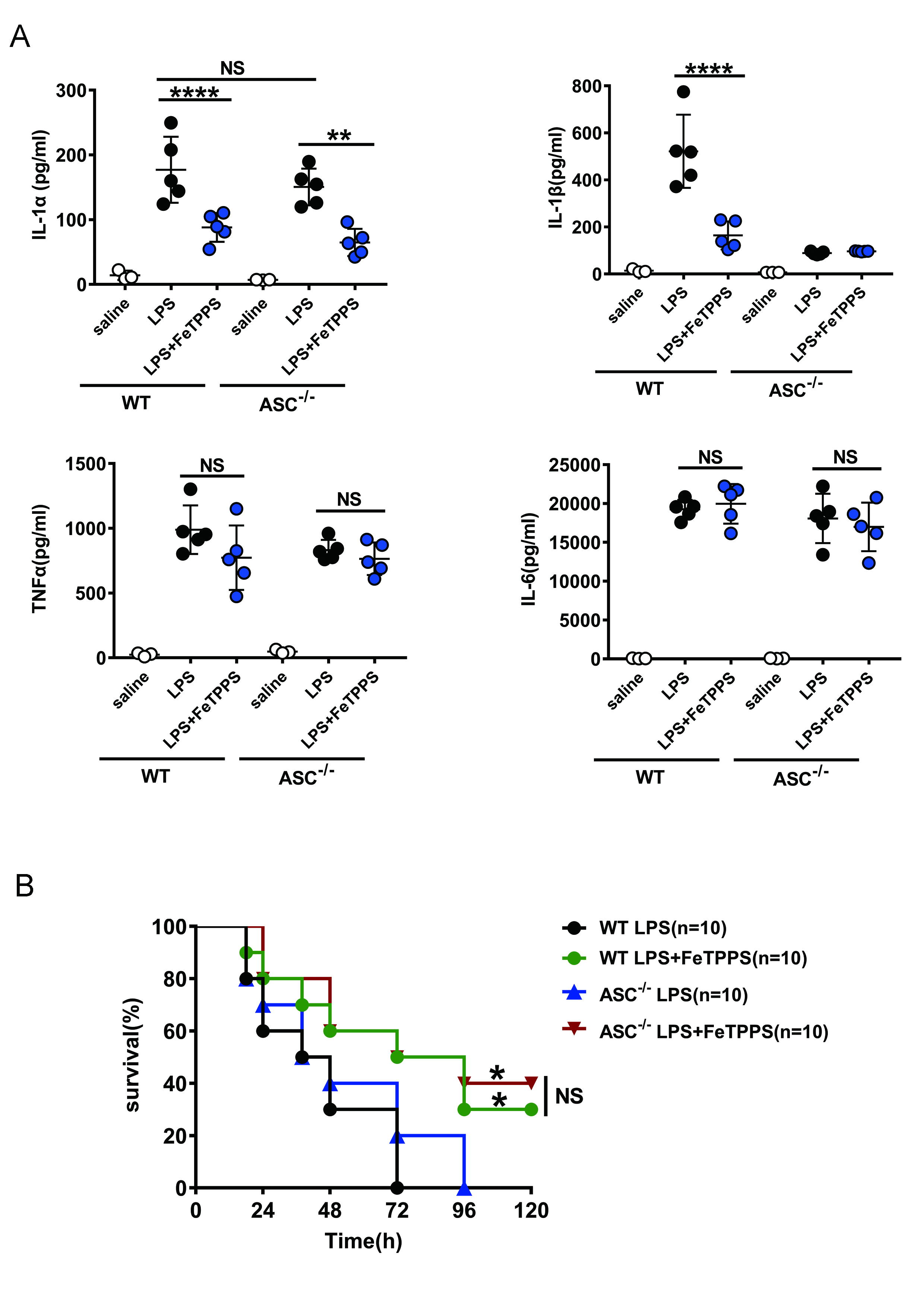

Supplement: Supplementary file 5 — FigS5 [file 41419_2021_3652_MOESM5_ESM.tif]

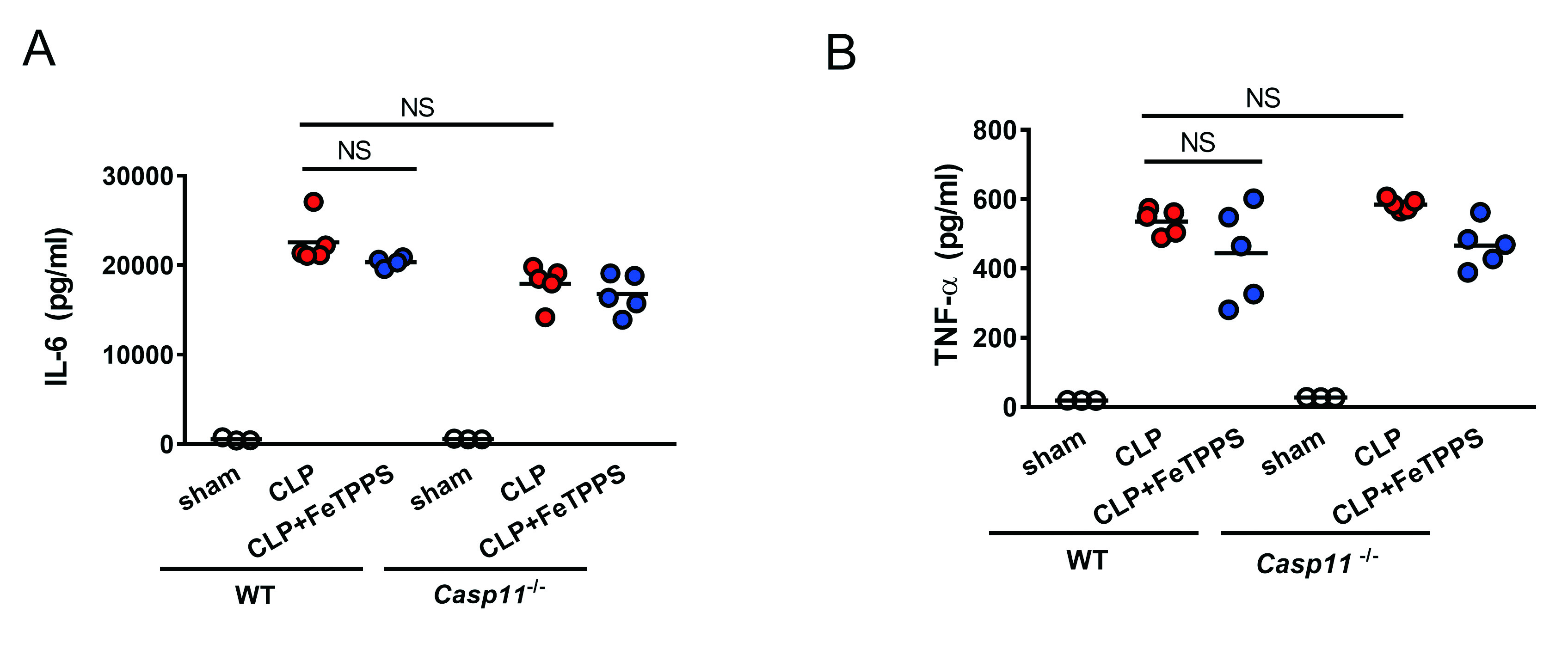

Supplement: Supplementary file 6 — FigS6 [file 41419_2021_3652_MOESM6_ESM.tif]

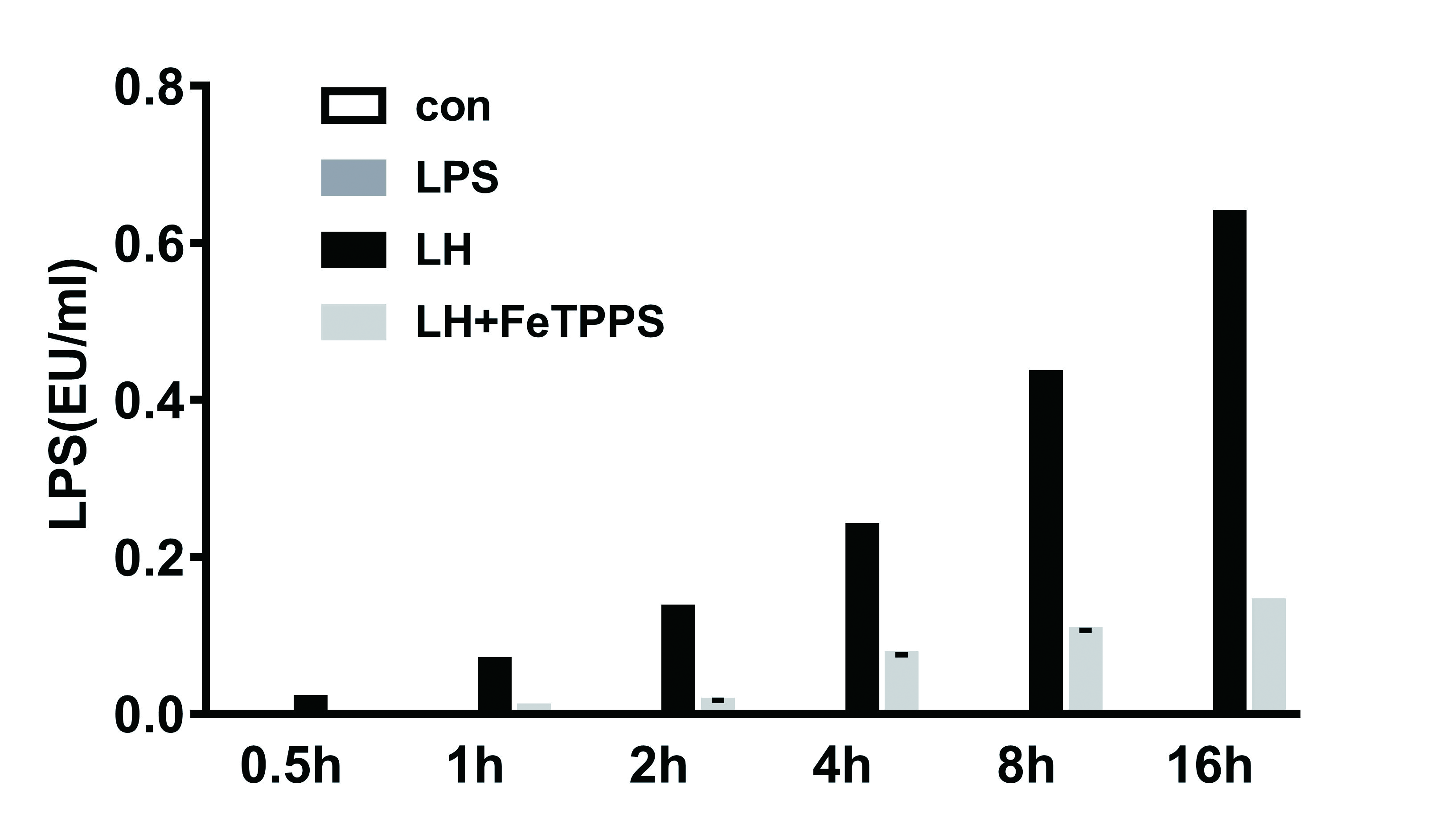

Supplement: Supplementary file 7 — FigS7 [file 41419_2021_3652_MOESM7_ESM.tif]

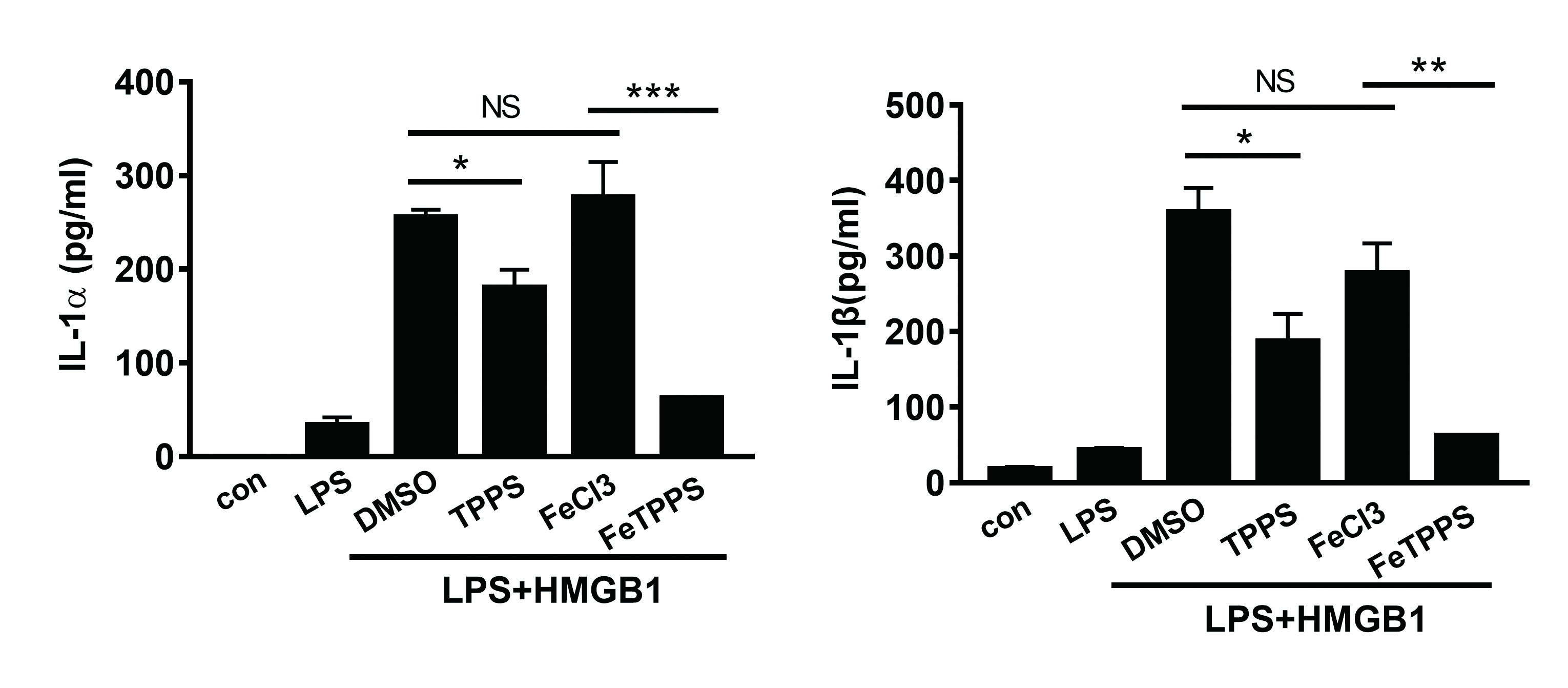

Supplement: Supplementary file 8 — FigS8 [file 41419_2021_3652_MOESM8_ESM.tif]

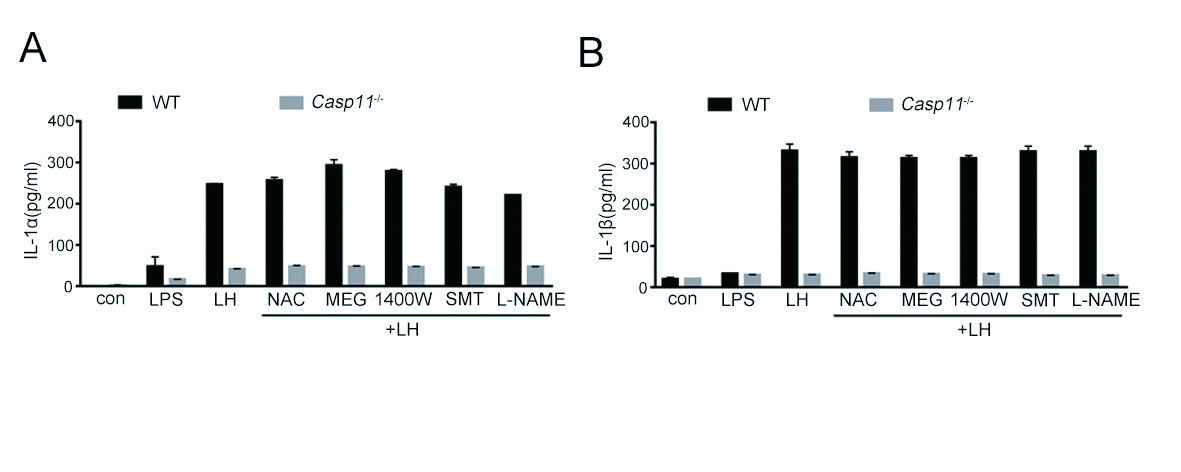

Supplement: Supplementary file 9 — FigS9 [file 41419_2021_3652_MOESM9_ESM.tif]

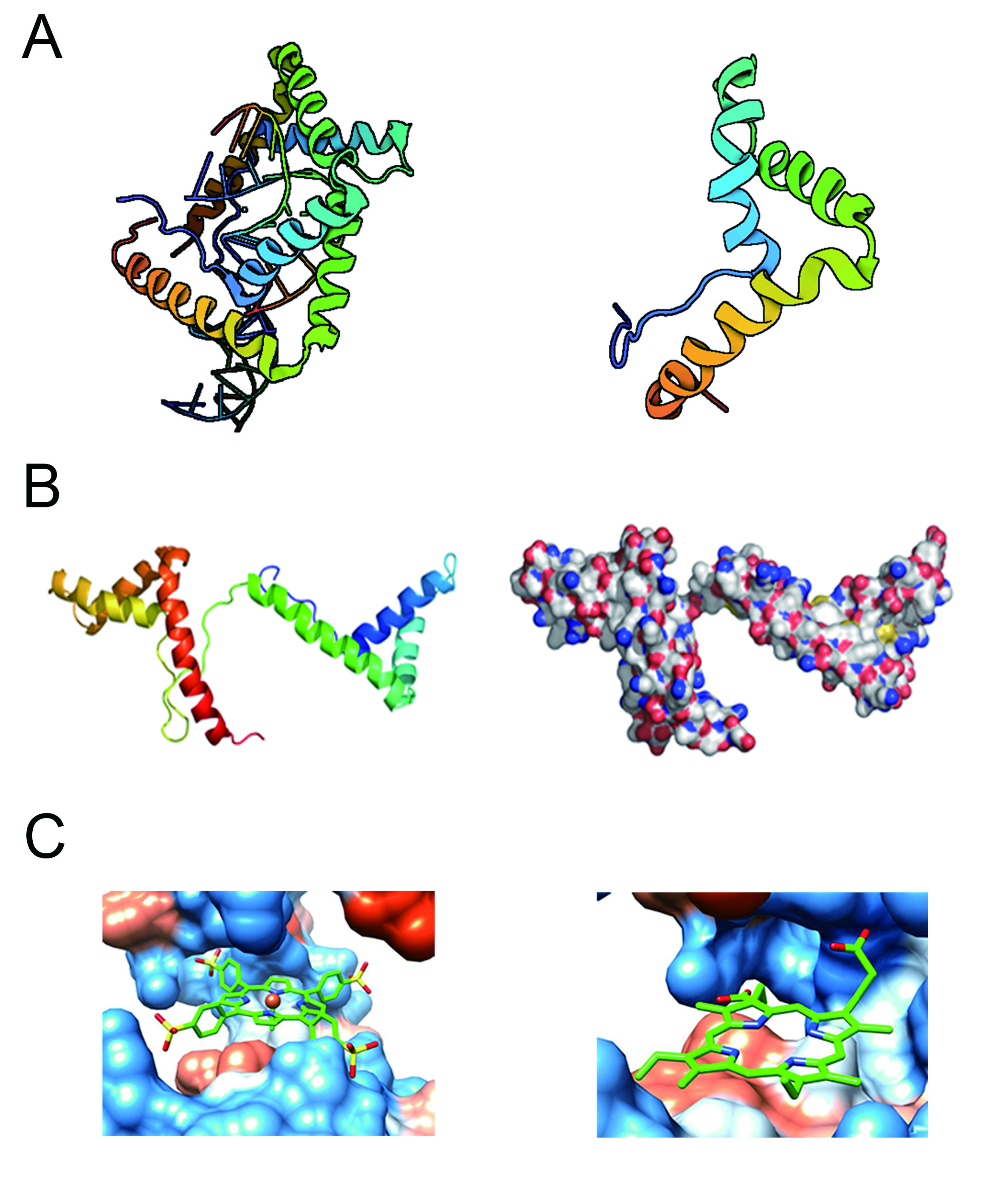

Supplement: Supplementary file 10 — FigS10 [file 41419_2021_3652_MOESM10_ESM.tif]

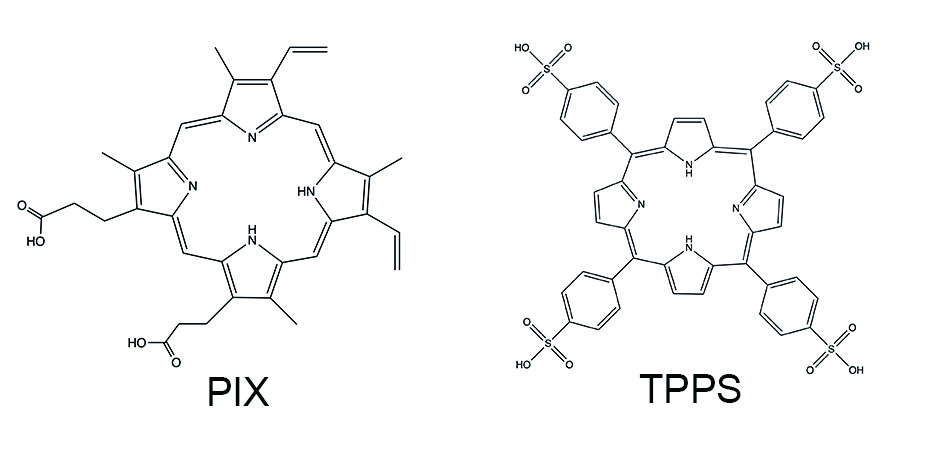

Supplement: Supplementary file 11 — FigS11 [file 41419_2021_3652_MOESM11_ESM.tif]
