# Supplementary material for: A small molecule binding HMGB1 inhibits caspase-11-mediated lethality in sepsis
Source: Cell Death Dis. 2021 Apr 14;12(4):402. doi: 10.1038/s41419-021-03652-5 (PMC8047024; doi:10.1038/s41419-021-03652-5)
Supplement: Supplementary file 12 — Supplementary informations [file 41419_2021_3652_MOESM12_ESM.docx]

**
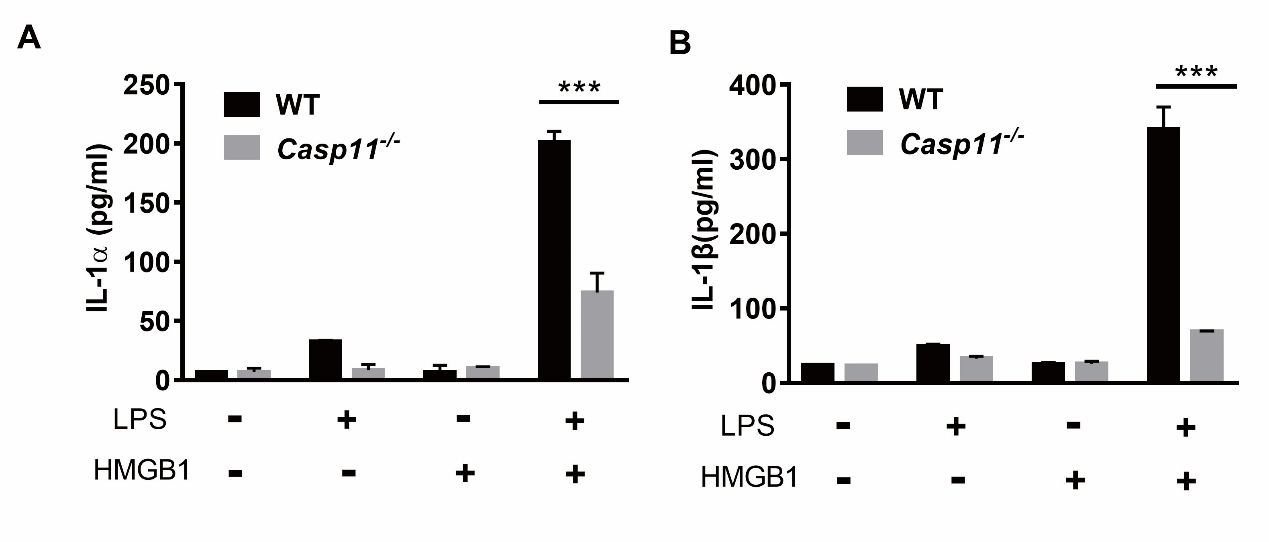
Supplementary Figures**

**Figure S1. HMGB1 Enabled Extracellular LPS to Activate Caspase-11**


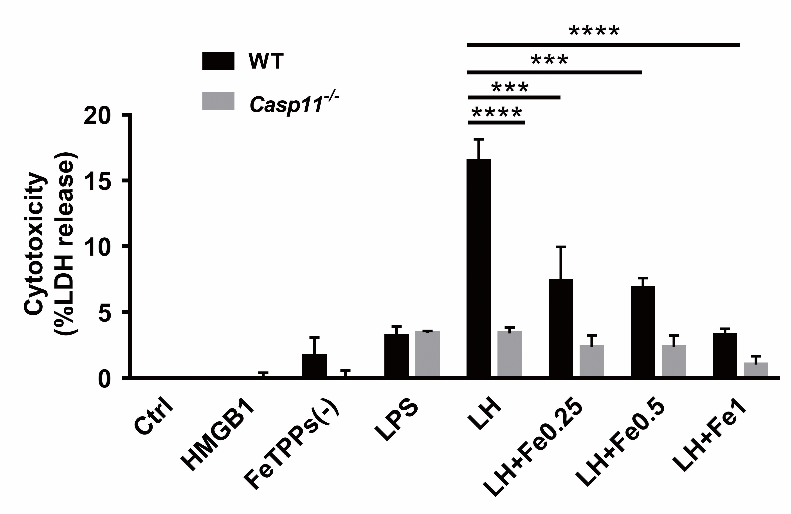
(A-B) Production of IL-1a and IL-1β release in the supernatants of WT or *Casp11^-/-^* peritoneal macrophages stimulated with LPS alone (1μg/mL) or LPS (1μg/mL)+HMGB1 (400ng/mL) for 16 h. Data are presented as mean ± SD of technical replicates. An unpaired *t*-test (two-sided) was used. IL-1α _WT VS Casp11_^-/-^ ****P*=0.008. IL-1β _WT VS Casp11_^-/-^ ****P*=0.002.

**Figure S2.** **FeTPPS inhibited HMGB1- and caspase-11-dependent LDH release *in vitro*.**

LDH release from WT or *Casp11^-/-^* peritoneal macrophages stimulated with LPS alone (1μg/mL) or LPS (1μg/mL) + HMGB1 (400ng/mL) in the absence or the presence of indicated concentrations of FeTPPS for 16h. Data presented as mean ± SD of technical replicates . Two-way ANOVA was used (_LH VS LH+FeTPPS 0.25μM/0.5μM /1μM_ ****P*=0.0002, ****P*=0.0001,*****P* <0.0001).

**
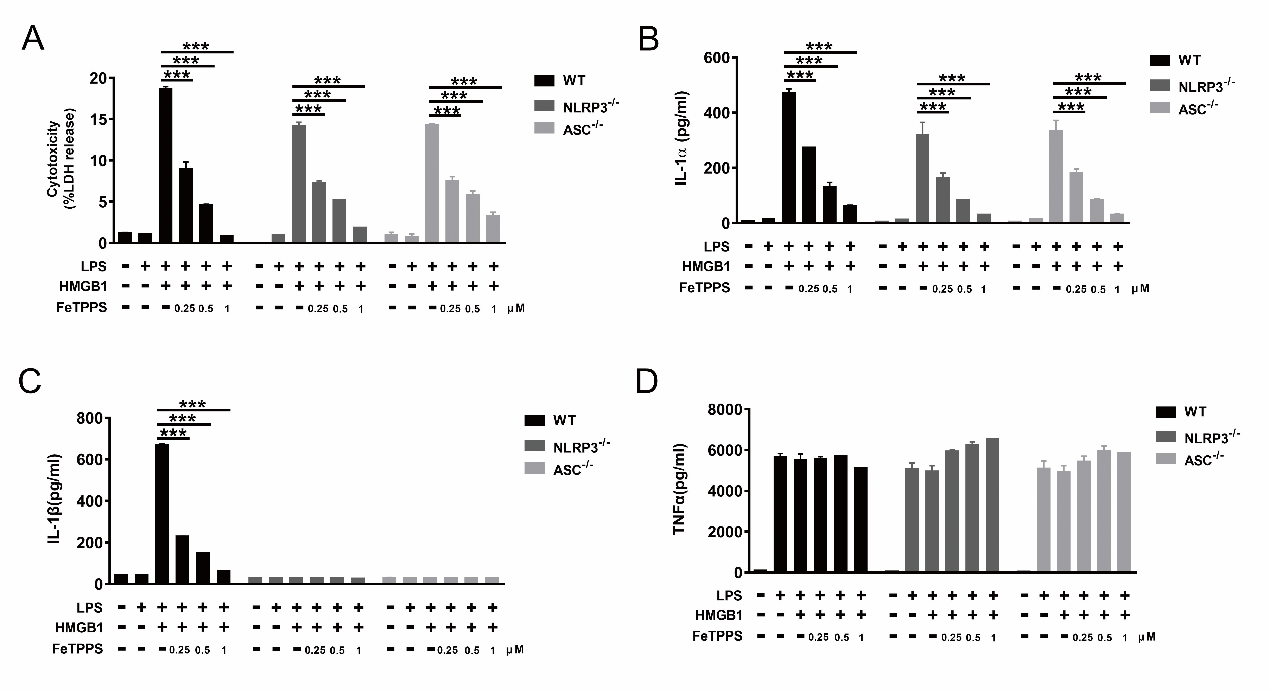
Figure S3. FeTTPS dose-dependently inhibited HMGB1+LPS-induced cytotoxicity and IL-1ɑ release in a manner independent of the NLRP3 inflammasome.**

**(A-D)** The release of LDH, IL-1α，IL-1β or TNFα from WT or *NLRP3****^-/-^*** or *ASC****^-/-^***  peritoneal macrophages stimulated with LPS alone (1μg/mL) or LPS (1μg/mL)+HMGB1 (400ng/mL) in the absence or the presence of indicated concentrations of FeTPPS for 16h. Data are presented as mean ± SD of technical replicates. Two-way ANOVA was used (_LH VS LH+FeTPPS 0.25μM/0.5μM /1μM_ ****P*<0.001).


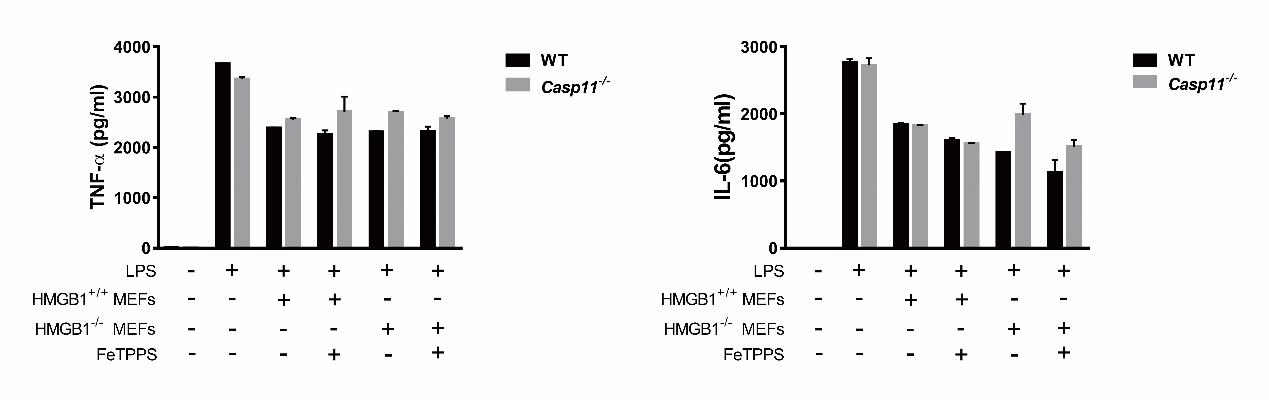
**Figure S4. FeTPPS did not inhibit TNFα and IL-6 release in HMGB1+LPS-stimulated macrophages.**

**(A-B)** Release of TNF-α and IL-6 from WT or *Casp11^-/-^* peritoneal macrophages stimulated with LPS alone (1μg/mL), LPS (1μg/mL)+HMGB1^+/+^ or HMGB1^-/-^MEF cells in the absence or the presence of FeTPPS(1μM) for 16h.

**
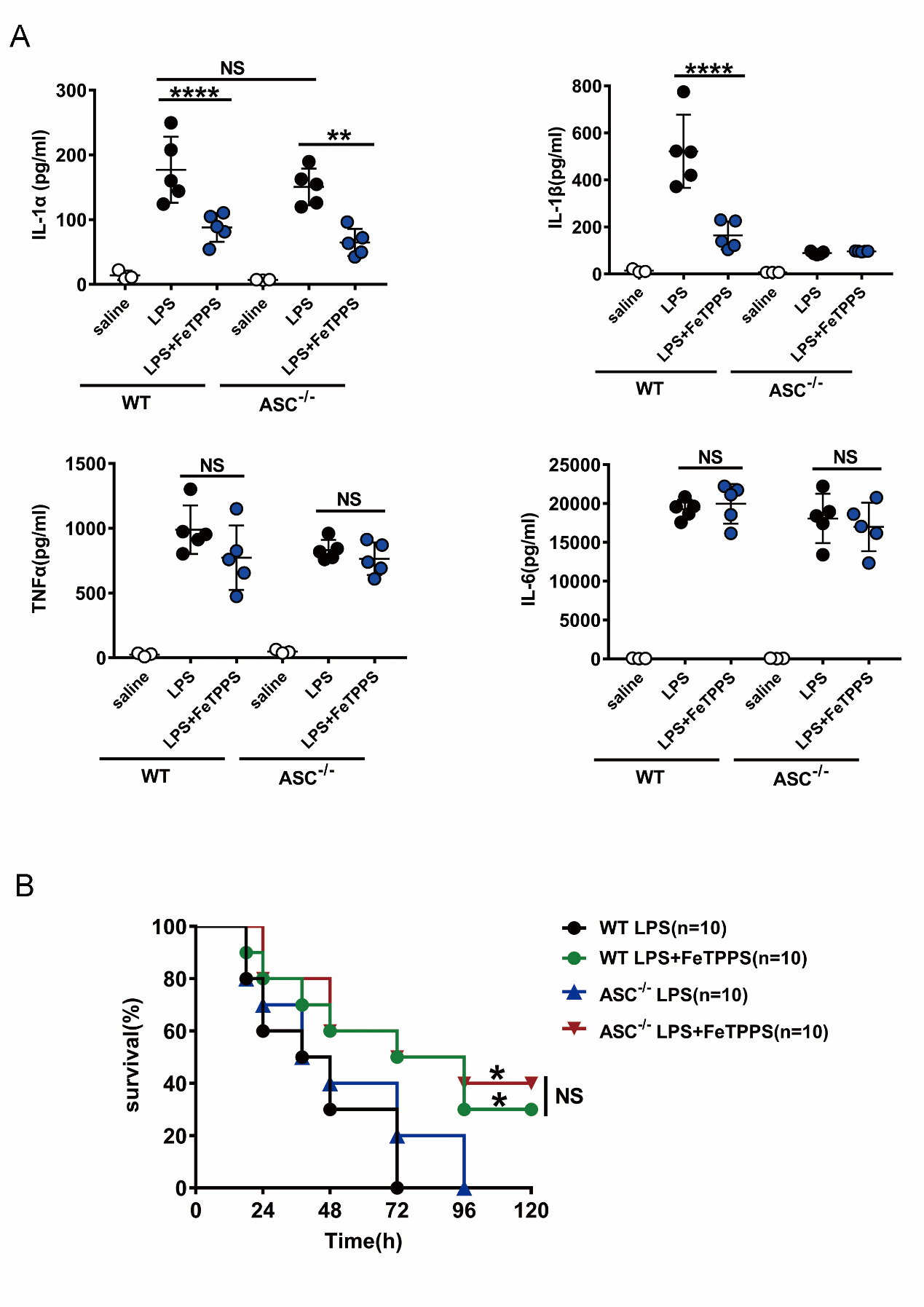
Figure S5. FeTPPS attenuated endotoxemia-induced IL-1α release and lethality in a manner independent of ASC.**

**(A)** WT or *ASC*^-/-^ mice pretreated with FeTPPS 6mg/kg or saline by intraperitoneal injection 1h before intraperitoneal challenge with 25mg/kg LPS. Serum levels of IL-1α, IL-1β, TNFα and IL-6 were measured. *****P*<0.0001，***P*<0.01.

**(B)** Kaplan Meier survival curves of WT or *ASC*^-/-^ mice pretreated with FeTPPS 6mg/kg by intraperitoneal injection 1h before intraperitoneal challenge with 25mg/kg LPS. **P*<0.05.


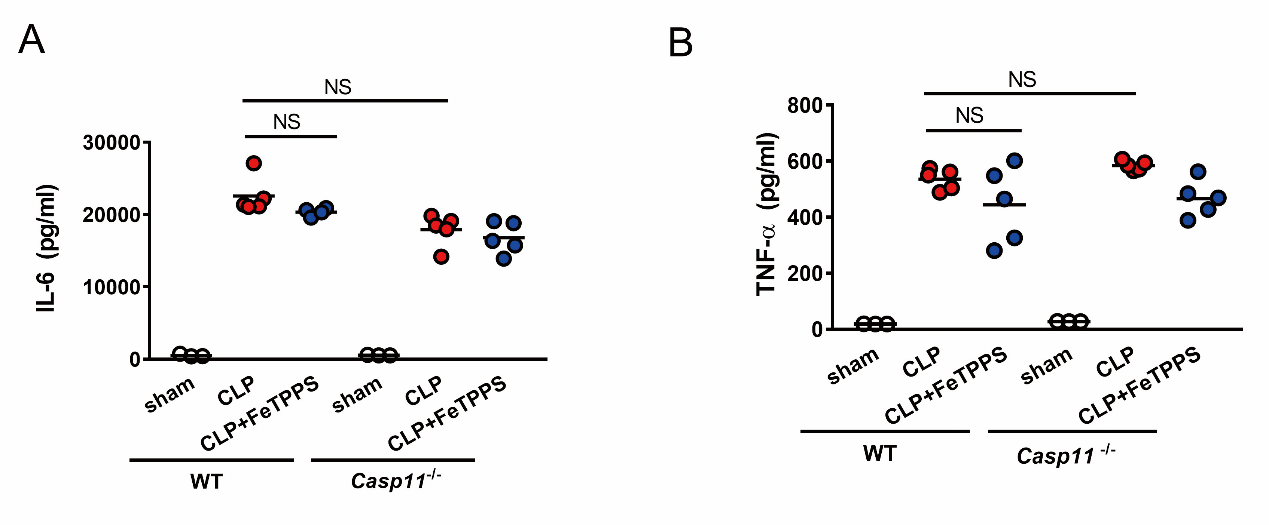


**Figure S6.** **FeTPPS treatment did not affect the release of serum IL-6 and TNFα in experimental sepsis.**

**(A-B)** WT or *Casp11^-/-^* mice were pretreated with FeTPPS 6mg/kg or saline by intraperitoneal injection 1h before subjected to cecum ligation and puncture (CLP) or sham operation. Serum levels of IL-6 and TNFα were measured by ELISA.


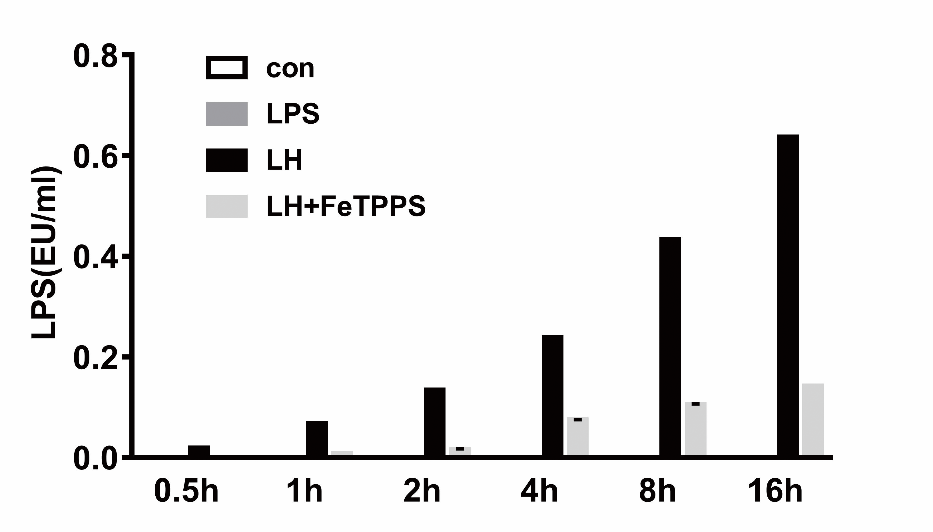


**Figure S7. FeTPPS inhibited HMGB1-mediated cytosolic delivery of LPS in a time-dependent manner.**

LPS (EU, endotoxin units) levels in the cytosolic fraction of mouse peritoneal macrophages at indicated time points as revealed by LAL assay.


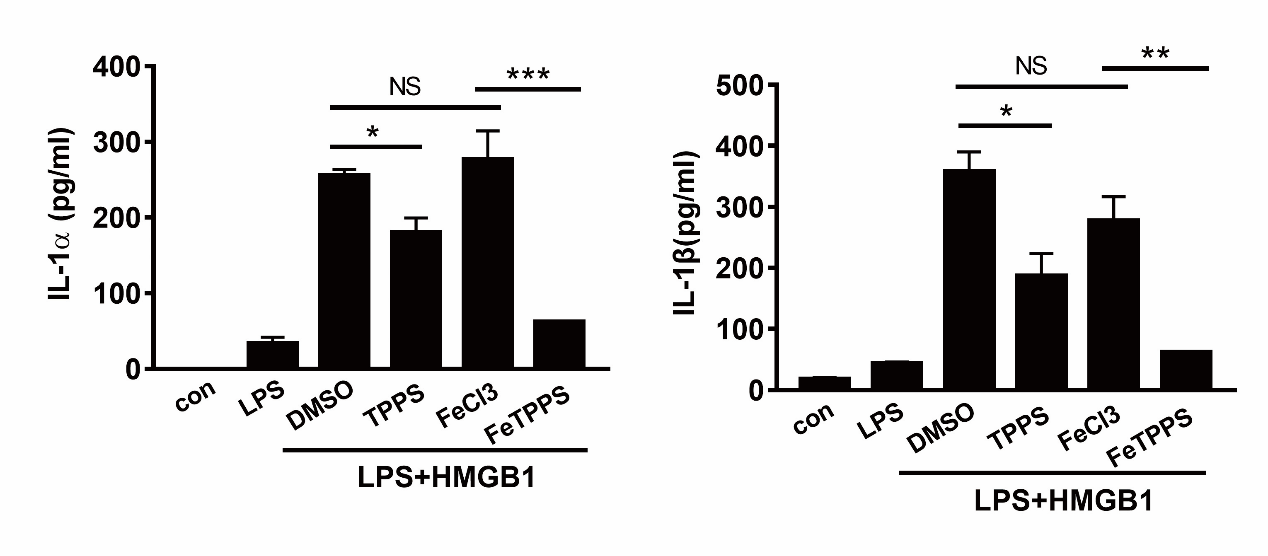
**Figure S8.** **FeTTPS suppresses HMGB1-mediated caspase-11 activation independent of Fe (III)**

(A-B) Release of IL-1α and IL-1β from mouse peritoneal macrophages stimulated with LPS (1μg/mL) + HMGB1 (400ng/mL) in the absence or the presence of indicated compounds for 16h. Data are presented as mean ± SD of technical replicates. An unpaired *t*-test (two-sided) was used. (IL-1α _DMSO VS TPPS_ **P*=0.0358, _DMSO VS FeTPPS_ ****P*=0.0009, IL-1β _DMSO VS TPPS_ **P*=0.0381, _DMSO VS FeTPPS_ ***P*=0.006).

**
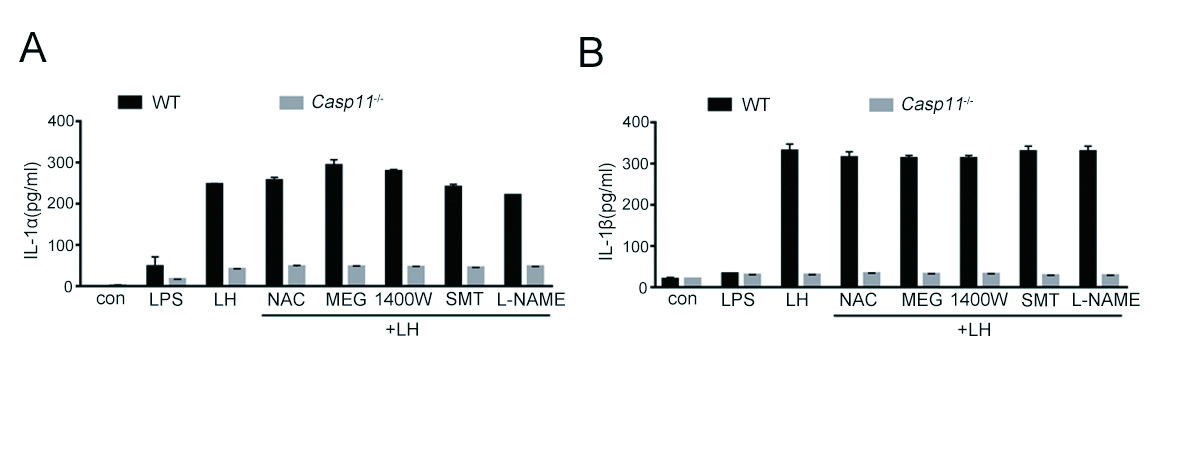
**

**Figure S9. Antioxidants except FeTPPS do not affect caspase-11 activation induced by HMGB1 and LPS**

**(A-B)** Release of IL-1α and IL-1βfrom WT or *Casp11^-/-^* peritoneal macrophages stimulated with LPS (1μg/mL) + HMGB1 (400ng/mL) in the absence or presence of indicated antioxidants for 16h.


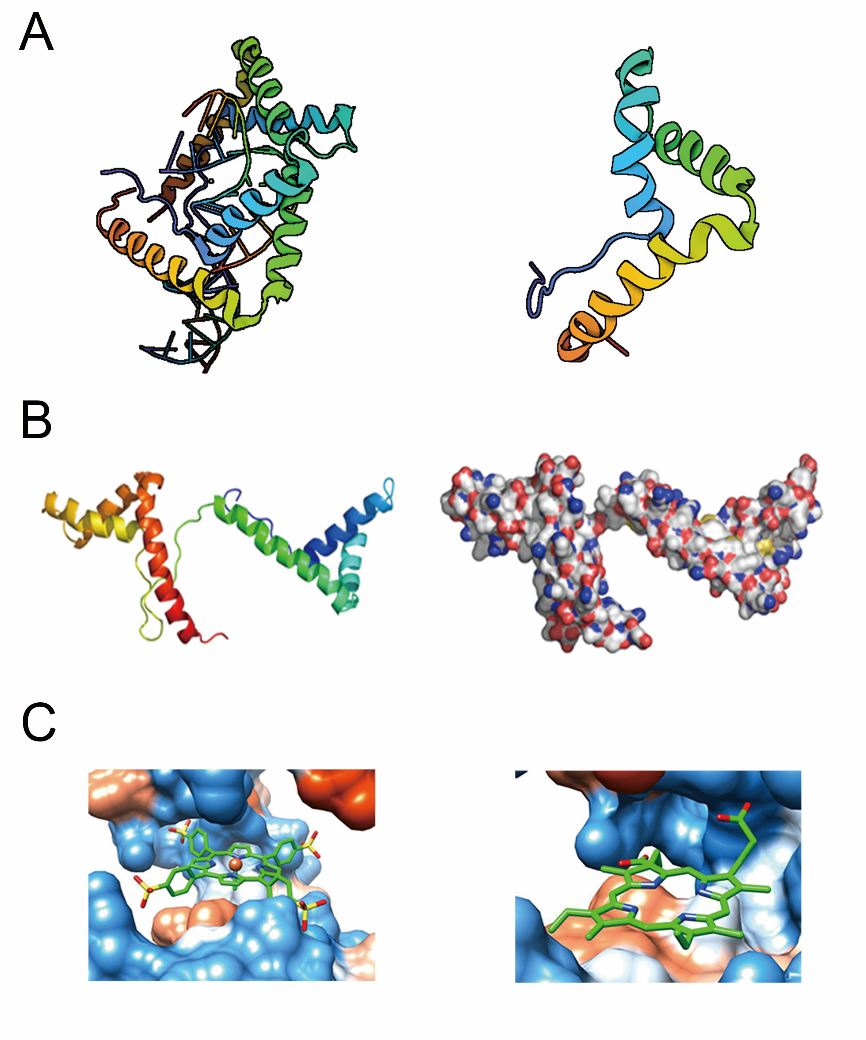


**Figure S10.** **HMGB1** **homologous modeling and docking poses.**

1. Crystal structure of Abox (PDB: 4QRP) (left panel) and Bbox (PDB:1HMF)(right panel) .
2. Mouse HMGB1 homologous modeling used by MODELLER program.
3. Flexible alignment showing the similar 3-D arrangement for low-energy conformations of

FeTPPS and HMGB1 (-10.88kcal/mol, left panel) or PIX and HMGB1 (-8.34kcal/mol, right panel).


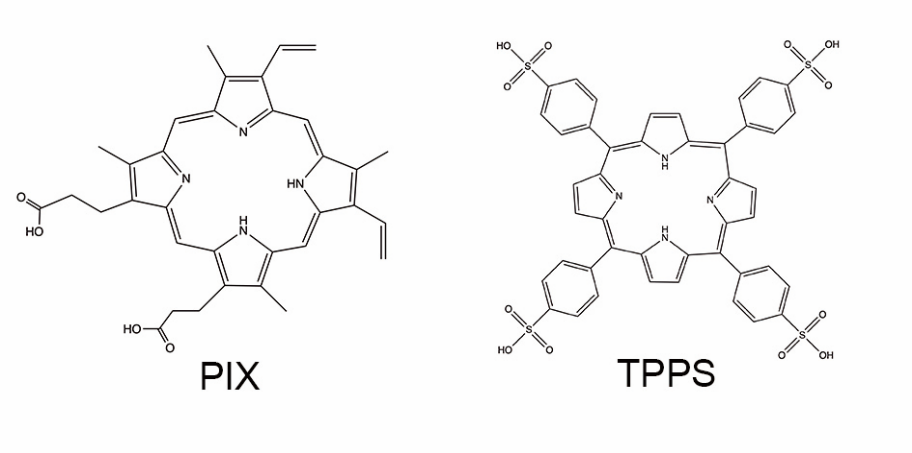


**Figure S11.** **Chemical structure of PIX, and TPPS.**

PIX: Protoporphyrin IX, TPPS: 5,10,15,20-Tetrakis(4-sulfophenyl) porphyrin.
